# Interfacial Charge Transfer Controls Plasmon-Enhanced Catalysis in DNA-Coated Gold Nanorods

**DOI:** 10.1021/acsnano.6c08886

**Published:** 2026-09-18

**Authors:** Bishal Pokhrel, Li Zuo, Tamie Vo, Pravin Pokhrel, Rebecca L. Stratton, Sajan Shakya, Adeola Adeyemi, Hanbin Mao, Wei-Shun Chang, Hao Shen

**Affiliations:** † Department of Chemistry and Biochemistry, 4229 Kent State University, Kent, Ohio 44242, United States; ‡ Advanced Materials and Liquid Crystal Institute, Kent State University, Kent, Ohio 44242, United States; § Department of Chemistry and Biochemistry, 205701 University of Massachusetts, Dartmouth, Dartmouth, Massachusetts 02747, United States

**Keywords:** chemical interface damping, plasmon-enhanced catalysis, gold nanorods, DNA corona, coronazyme, long-range charge transport

## Abstract

The ultrafast recombination of plasmon-generated hot carriers (within ∼100 fs) has long prevented their productive use in catalysis. Here, we show that a DNA corona coated on gold nanorods (AuNRs) overcomes this barrier through chemical interface damping (CID): hot electrons generated by longitudinal plasmon excitation transfer directly into the DNA layer, creating charge-separated states that drive long-range catalysis within the corona phase. Using three complementary single-particle techniques, single-particle force spectroscopy, single-particle scattering spectroscopy, and single-particle fluorescence microscopy, we establish the first quantitative, sample-level correlation between population-averaged interfacial charge transfer efficiency (η_CT_) and enzyme-like catalytic activity. As the DNA:AuNR feed ratio increases, η_CT_ rises from 20% to 32%, and the plasmon-enhanced turnover rate increases proportionally. Photothermal effects are rigorously excluded by control experiments. These results reveal that the DNA corona simultaneously serves as a substrate-binding scaffold and stabilizes interfacial charge-separated states, establishing CID engineering as a design principle for next-generation plasmonic photocatalysts.

## Graphical abstract

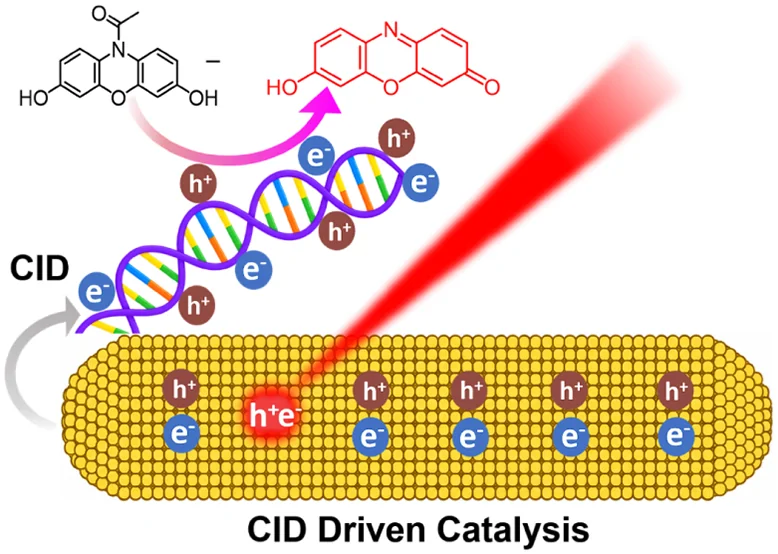

## Introduction

The directed conversion of photon energy into chemical work represents one of the central ambitions of modern catalysis. Plasmonic nanoparticles, particularly gold nanorods (AuNRs), are compelling candidates for this role: their strong localized surface plasmon resonance (LSPR) enables efficient visible-light absorption, and plasmon decay generates highly energetic hot carriers capable of driving chemical transformations that would otherwise require elevated temperatures or strong chemical reductants.^1–7^ Yet, utilizing this potential has proven difficult. The lifetime of plasmon-generated hot carriers is controlled by competing ultrafast relaxation pathways, including electron-electron scattering and electron-hole recombinations less than 100 fs.^8–15^ This time scale is orders of magnitude shorter than the microsecond-to-millisecond time scales of most catalytic turnovers, leaving only a small fraction of hot carriers available for productive chemistry.^16,17^ Extending hot carrier lifetime or equivalently creating a charge-separated state that persists long enough to reach a reactive substrate is therefore the decisive challenge in plasmonic photocatalysis.

Chemical interface damping (CID) has emerged as a mechanistically distinct pathway for hot carrier extraction that partially circumvents the recombination problem.^17–22^ Unlike indirect hot electron transfer, in which a plasmon decays into a high-energy electron that must overcome the energy barrier between metal/semiconductor or metal/molecule interfaces before injection into an adjacent molecule, CID involves direct plasmon decay into an interfacial charge transfer state between the metal and a chemisorbed molecule. This direct mechanism outcompetes the ultrafast relaxation pathways of plasmon decay and manifests experimentally as a broadening of the homogeneous LSPR line width, because the interfacial electron transfer shortens the plasmon dephasing time. Although CID has been probed in the time domain using ultrafast spectroscopy,^17,23^ single-particle dark-field spectroscopy has been the alternative tool for detecting and quantifying CID in the frequency domain,^24–29^ since ensemble measurements obscure the intrinsic line width behind inhomogeneous broadening arising from nanoparticle size and shape polydispersity.^30,31^ CID has been demonstrated in AuNRs functionalized with thiols,^25^ small molecules,^27,32^ and oligonucleotides;^28^ however, the separated charge could recombine at the Au/molecule junction, leading to a shorter hot-carrier lifetime and lower photocatalytic activity.^17^ Therefore, it is critical to quantitatively connect CID to the catalytic turnover of the hybrid photocatalysts. Whether stronger CID produces more efficient catalysis, and to what degree, has remained an open question.

We recently introduced the coronazyme as a new class of artificial enzyme that integrates the functions of a nanozyme core and a DNA corona into a single, synergistic catalytic platform.^33^ In the original AuNS@DNA coronazyme, a DNA hairpin containing poly­(adenosine) internal loops coats a 5 nm gold nanosphere (AuNS), and we demonstrated that catalysis occurs not at the metal surface but at reactive sites within the DNA corona, driven by radical transport from the AuNS through the DNA strand to substrate molecules bound at remote bases. The Au core serves as the radical initiation center, while the DNA corona functions both as a radical/charge transporter and provides substrate binding sites. This long-range mechanism depends on charge transfer along the DNA backbone, a process independently confirmed in DNA-bridged plasmonic systems where hot electrons generated by silver nanoparticles travel several nanometers through a double-stranded DNA wire to drive a hydrodehalogenation reaction at a distal site.^34^ These findings collectively imply that the DNA corona is not simply a substrate-binding scaffold; it is an intrinsic charge transport medium. If the initial charge injection from the AuNR into the DNA through the CID step can be made more efficient, the entire catalytic chain should accelerate accordingly.

Here, we test this hypothesis directly. We replace the AuNS nanocore with a gold nanorod, whose anisotropic geometry produces a well-separated longitudinal LSPR in the near-infrared that can be selectively excited without triggering interband transitions,^35^ enabling clean isolation of the CID-driven catalytic pathway. Employing three complementary single-particle techniques on the same system, specifically single-molecule force spectroscopy (SMFS) to confirm DNA binding and corona formation, single-particle scattering spectroscopy (SPSS) to measure CID efficiency at the individual nanoparticle level, and single-particle fluorescence microscopy (SPFM) to quantify catalytic turnover kinetics, we establish the first direct, sample-level correlation between population-averaged CID efficiency and population-averaged catalytic enhancement between interfacial charge transfer efficiency and enzyme-like catalytic activity. The DNA corona systematically increases CID efficiency as DNA:AuNR feed ratio increases, and this enhancement maps quantitatively onto the observed catalytic rate improvement. Bare AuNRs cannot sustain long-lived charge-separated states at their citrate-coated surfaces and show no light-induced catalytic enhancement. The AuNR@DNA system thus functions as a photochemically gated catalyst: plasmon excitation injects charges into the DNA corona via CID, the DNA transports those charges to substrate-bound reactive sites, and turnover proceeds. This charge transduction chain provides a rational, mechanistically grounded design principle for next-generation plasmonic photocatalysts built with programmable biomolecular coatings.

## Results and Discussion

### Construction of the DNA-Coated AuNR System (AuNR@DNA)

As poly-deoxyadenosine can bind to the Au surface,^36,37^ we designed a DNA stem-loop structure that contained two poly­(dA)_21_ segments as internal loops connecting two duplex DNA stems (Figure 1A, for sequence and preparation please see Table S1 and Figure S1). First, we performed SPFS to reveal the unfolding features of this DNA oligo without AuNR. To this end, the construct was tethered between optically trapped beads via biotin-streptavidin and digoxigenin/anti-digoxigenin linkages using 1558-bp and 2391-bp dsDNA handles (see Figure 1A and Supporting Information Sections S2–S4). When the two optically trapped beads were separated by steering one of the bead-trapping lasers away from another, the lower dsDNA stem together with the two poly­(dA)_21_ internal loops was unfolded first (step (1) in Figure 1B), followed by the upper dsDNA stem and the terminal loop (step (2) in Figure 1B). The rupture forces for both features were centered at 12.9 pN (Figure 1D and E), consistent with unzipping forces for duplex DNA.^38,39^ As shown in Figure 1F, the change-in-contour-length (Δ*L*) histogram revealed two populations corresponding to step 1 and step 2 unfolding events (for step 1, Δ*L* = 36.5 nm, expected Δ*L* = 39.6 nm, see Figure S4C; for step 2, Δ*L* = 15.4 nm, expected Δ*L* = 22.3 nm, see Figure S4C). The small discrepancy for the terminal hairpin likely reflects end-fraying of duplex DNA stems in stem-loop structures.^39^

**Figure 1:**
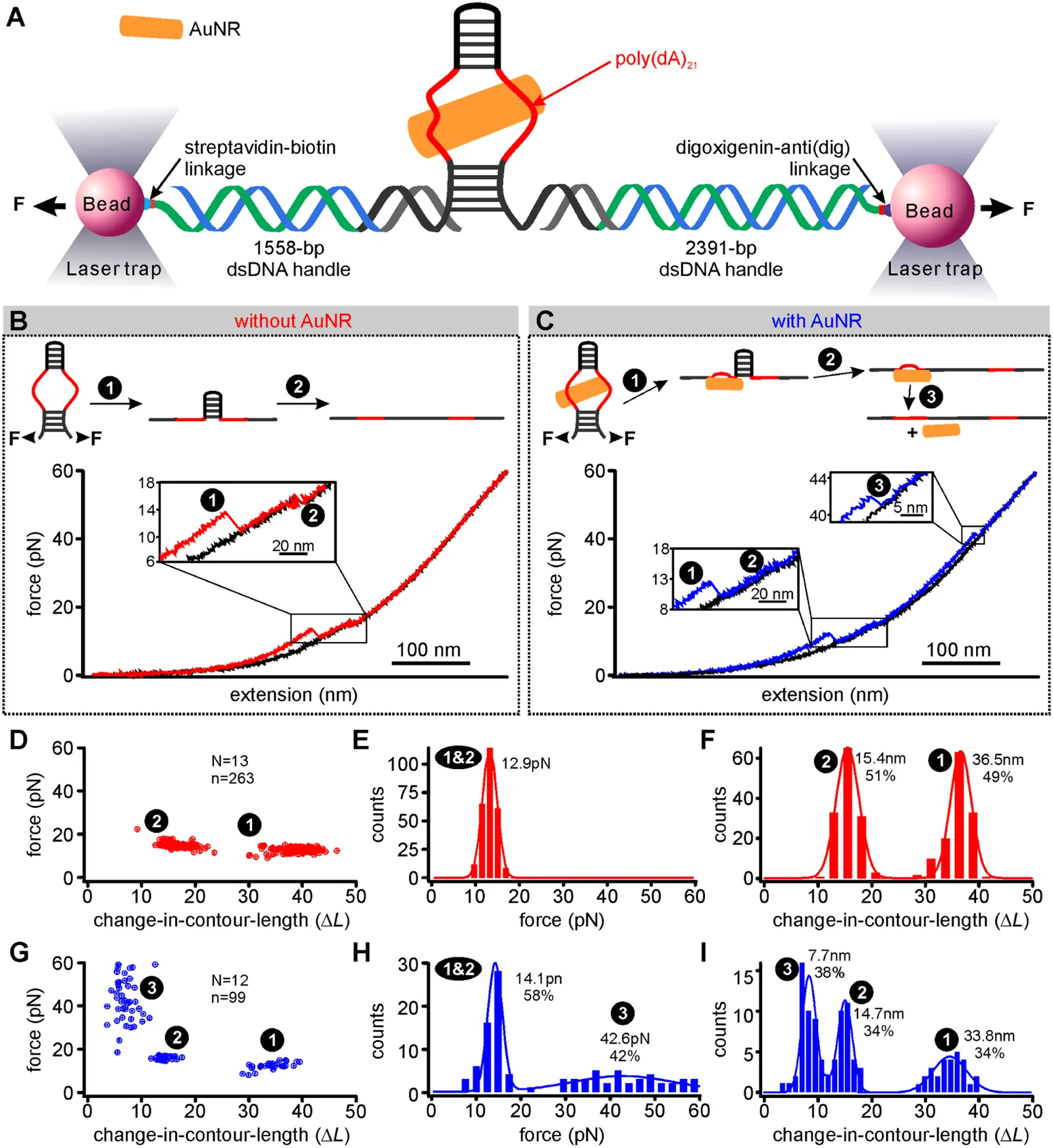
Confirmation of DNA coated AuNR. (A) Schematic of the single-particle force spectroscopy to probe the interaction between DNA hairpin and AuNR. A typical force–extension (FX) curve when the DNA construct is stretched (red) and relaxed (black) in the absence (B) and in the presence (C) of AuNR in the optical tweezers instrument. The insets represent rupture features marked with numbers that correspond to unfolding pathway. (D, G) Scatter plot of unfolding force vs change-in-contour length obtained from those FX curves in the absence and presence of AuNR, respectively. “N” represents number of molecules investigated, and “n” represents total number of FX traces. (E, H) Rupture force histograms and (F, I) change-in-contour-length histograms of the unfolding features. Solid curves represent Gaussian fittings of the histograms.

To ensure AuNR binds to the DNA construct, next, we incubated the DNA construct with AuNRs (diameter:length = 18:52 nm; aspect ratio = 2.80 (Figure S3 in the Supporting Information); DNA:AuNR feed ratio 10:1) at −80 °C for 15 min, followed by thawing at room temperature. Such a freeze-thaw procedure allowed poly­(dA)-mediated Au–N interactions at the poly­(dA)_21_ internal loop regions.^39^ The poly­(A) segments are essential for stable AuNR functionalization through the adenine-Au interaction; alternative sequences exhibit substantially weaker surface affinity and are susceptible to salt-induced aggregation at the DNA feed ratios used here.^39,40^ Mechanical unfolding of the AuNR-bound DNA construct revealed a new high-force rupture feature (step 3) absent in the pure DNA control (compare Figure 1C and B). This feature exhibited a mean rupture force of 42.6 pN, substantially higher than those for the steps (1) and (2). In addition, it showed a mean Δ*L* of 7.7 nm – far smaller than those of the two previous steps. The elevated rupture force and reduced Δ*L* are consistent with the binding of AuNR by the two poly­(dA)_21_ internal loops, which resists mechanical unfolding at lower force while producing a much reduced Δ*L* as a result of straightening poly­(dA)_21_ segments due to the ejection of AuNR. Previously, similar 3-step features have been observed during the unfolding of the same DNA construct bound with a 5 nm AuNS.^39^

### Measurement of CID at AuNR-DNA Interface by Single-Particle Scattering Spectroscopy

With AuNR@DNA formation confirmed at the single-molecule level, we next quantified the CID effect that arises from the Au-DNA interface. CID directly controls the efficiency with which plasmon excitation injects hot electrons into the DNA corona and thus governs the degree to which longitudinal LSPR excitation can enhance catalytic turnover. SPSS was used to measure the homogeneous LSPR line width of individual AuNRs before and after DNA functionalization, providing a direct, particle-resolved measure of the interfacial charge transfer efficiency η_CT_ for each DNA:AuNR feed ratio condition.

To directly quantify the charge transfer at the AuNR-DNA interface, we measured scattering spectra of individual AuNRs and AuNR@DNAs using a custom dark-field hyperspectral microscope (Figure 2A, B, Experimental Section and Supporting Information S5). CID manifests as a broadening of the homogeneous LSPR line width *Γ*, which corresponds to an LSPR dephasing time of less than 10 fs.^31^ This additional line width broadening may arise from either interfacial electron transfer or plasmon-induced resonance energy transfer on this ultrafast time scale.^41,42^ However, the UV absorption spectrum of DNA does not overlap with the LSPR band in the visible region, whereas such spectral overlap is required for plasmon-induced resonance energy transfer. Therefore, the observed line width broadening is attributed primarily to interfacial electron transfer: when hot electrons transfer from the AuNR into the LUMO of adsorbed DNA bases upon plasmon excitation, the coherent oscillation of conduction electrons is disrupted, shortening the plasmon dephasing time and widening the spectral line width.^24–29^

**Figure 2:**
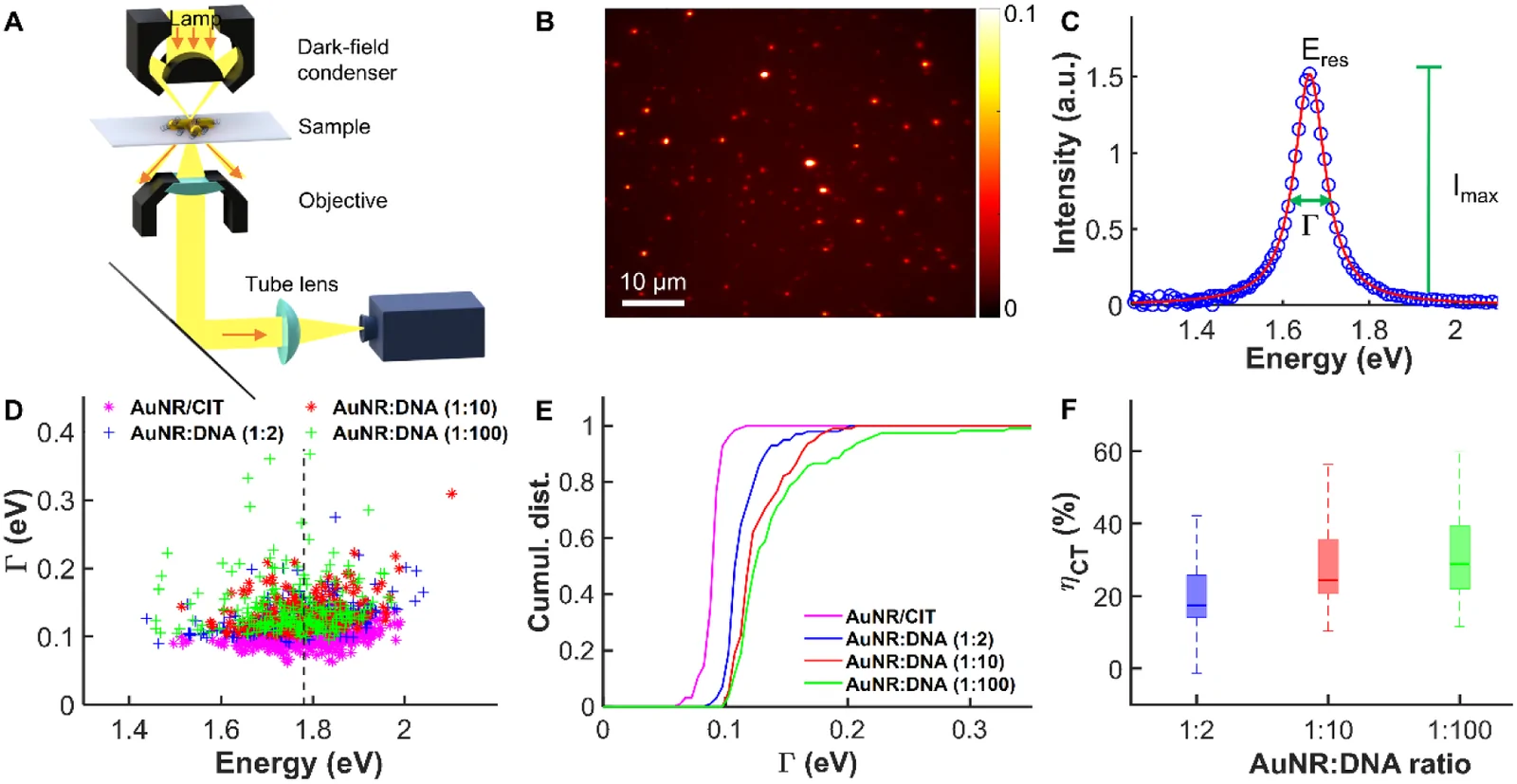
(A) Schematic of the single-particle dark-field hyperspectral microscopy setup. (B) Hyperspectral image of sparsely dispersed AuNR@DNA; color depth variation reflects particle-to-particle heterogeneity in scattering intensity. (C) Representative scattering spectrum of a single AuNR (circles) fit to a Lorentzian function (red curve). (D) Scatter plot of E_res_ vs. Γ for citrate-capped AuNRs and AuNR@DNA at varying DNA:AuNR feed ratios; each symbol represents one particle. The dashed line represents E_res_ = 1.78 eV. The broader Γ distribution and increased number of outlier data points at the 100:1 DNA:AuNR feed ratio reflect greater particle-to-particle heterogeneity in DNA surface density at higher feed ratios, arising from the stochastic nature of poly­(A) adsorption when DNA is present in large excess; this heterogeneity would be obscured in ensemble measurements. (E) Cumulative distribution of Γ for each sample. The cumulative distribution represents the fraction of AuNRs with a value lower than the one indicated in the *x*-axis. (F) Box plot of η_CT_ for AuNR@DNA at DNA:AuNR feed ratios of 2:1, 10:1, and 100:1.

This broadening is resolvable only at the single-particle level, since ensemble measurements average over the broad size and shape distributions of colloidal AuNRs, masking the intrinsic homogeneous line width behind inhomogeneous broadening.^30,31^ Lorentzian fitting of single-particle scattering spectra yielded the resonance energy *E*_res_ and line width *Γ* for hundreds of individual particles (Figure 2C, D). To exclude contributions from interband transitions, only particles with *E*_res_ < 1.78 eV were included in the analysis. As-received citrate-capped AuNRs exhibited a mean *Γ* of 91 ± 8 meV (n = 124 particles). The intrinsic variation among the AuNR@DNA samples is visible in Figure 2D, which is one of the advantages of single-particle analysis, as ensemble analysis would average out such differences. Upon DNA functionalization, *Γ* broadened systematically with increasing DNA:AuNR feed ratio: 116 ± 16 meV at DNA:AuNR = 2:1 (n = 99 particles), 129 ± 23 meV at 10:1 (n = 95 particles), and 142 ± 43 meV at 100:1 (n = 112 particles) (Figure 2E). The charge transfer efficiency was quantified as ηCT=ΓCTΓ , where Γ_CT_ is the CID-attributed line width increment relative to bare AuNR, yielding η_CT_ values of 20 ± 12%, 28 ± 13%, and 32 ± 15% for DNA:AuNR feed ratios of 2:1, 10:1, and 100:1, respectively (Figure 2F). The measured line width broadening reflects the CID contribution *Γ*_CID_, isolated by subtracting the mean line width of bare AuNRs, which captures the combined contributions of radiative damping, electron surface scattering, and electron-electron scattering, from the line width of each individual AuNR@DNA.^24,29^ Control experiments using poly­(A)-based constructs with varying numbers of Au-binding arms but identical DNA hairpin bodies confirm that the CID-induced line width broadening scales with the number of adenine-Au contacts rather than with the overall DNA mass added to the nanorod surface, ruling out a refractive index origin for the observed *Γ* increase. To further exclude local refractive index variations from the line width broadening, we calculated scattering spectrum for an adsorbed coating layer across a refractive index range of *n* = 1.33 (PBS buffer, no coating) to *n* = 1.50 (DNA coating, full coverage) using established models^25^ (Figure S7 in the Supporting Information). These calculations reveal that the increase of local dielectric constant from 1.33 to 1.50 reduces the plasmon line width by less than 1 meV, which cannot account for the experimentally observed 25–51 meV broadening. Therefore, we can conclude that CID is the dominant mechanism driving the plasmon line width change upon DNA adsorption.

### Peroxidase-Like Catalytic Kinetics of the AuNR@DNA Coronazyme

The peroxidase-mimicking activity of individual AuNR@DNA was characterized by SPFM using the fluorogenic substrate Amplex Red (AR) (Figure 3A). The system demonstrates no catalysis without Au or with DNA alone.^33^ In presence of H_2_O_2_, AuNR catalyzes the oxidative conversion of AR into the highly fluorescent product resorufin (RF, maximum emission ∼583 nm) (Figure 3B). Single-particle catalysis was performed inside a microfluidic chamber in which the nanoparticles were immobilized on the coverslip surface and exposed to a continuous flow of reaction mixture. RF generation events appeared as recurrent, spatially-fixed fluorescence bursts, and super-resolution localization (∼4 nm precision) was used to assign each burst to its parent nanoparticle (Figure 3C). The inter-burst waiting time τ, defined as the interval between two consecutive RF formation events on a single catalyst, was extracted and statistically analyzed to yield the intrinsic catalytic properties of each individual particle.

**Figure 3:**
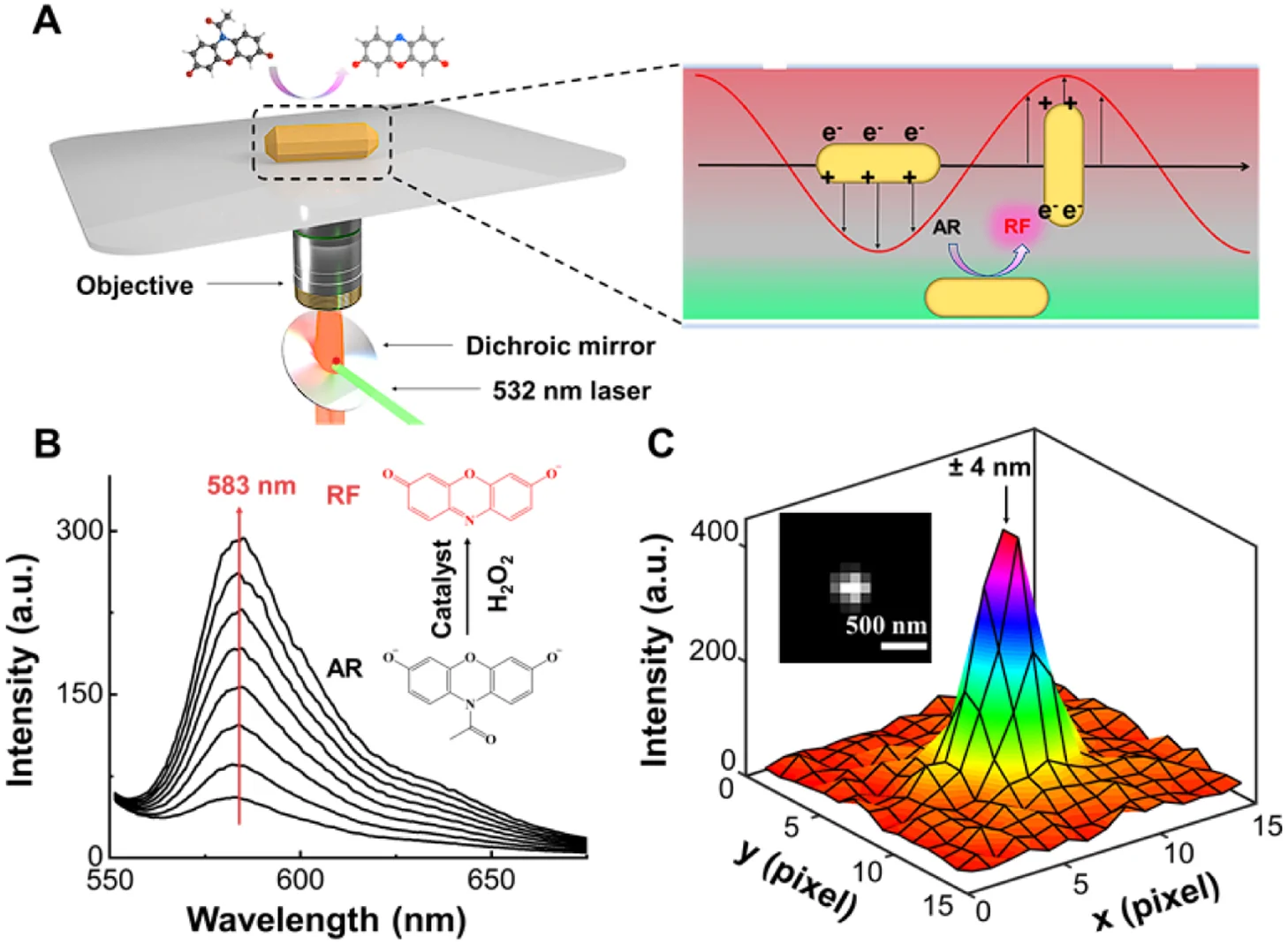
(A) Scheme of the single-particle catalysis experiments. AuNR@DNA are sparsely dispersed on a glass coverslip, while the reactant mixture at various concentrations (AR and H_2_O_2_) flows continuously to maintain steady-state conditions. A 532 nm laser excites RF products generated on individual particles; an external LED excites the longitudinal LSPR of AuNRs; and an sCMOS camera captures fluorescence emission after spectral filtering. (B) Ensemble fluorescence spectra showing the progressive increase in RF emission at 583 nm as AR is oxidized by AuNR in the presence of H_2_O_2_. (C) Super-resolution localization of a single RF molecule by two-dimensional Gaussian fitting of its emission spot, with a localization precision of ∼4 nm.

The time-averaged turnover rate ⟨τ⟩^–1^ increased with AR concentration and saturated at high concentrations (Figure 4A, black), consistent with modified Michaelis–Menten kinetics:^33^

1 $${\langle \tau \rangle }^{-1}=\frac{V_{\max}\left[S\right]}{K_{M}+\left[S\right]}$$

in which *V*_max_ represents the highest possible reaction rate for a single catalyst, *K*_M_ the Michaelis–Menten constant reflecting the binding affinity of substrate, and [*S*] the substrate concentration. For bare AuNRs, *V*_max_ = 0.050 ± 0.002 s^–1^ and *K*_M_ = 0.24 ± 0.02 μM, giving a catalytic efficiency (*V*_max_ /*K*_M_) of 0.21 ± 0.03 s^–1^ μM^–1^.

**Figure 4:**
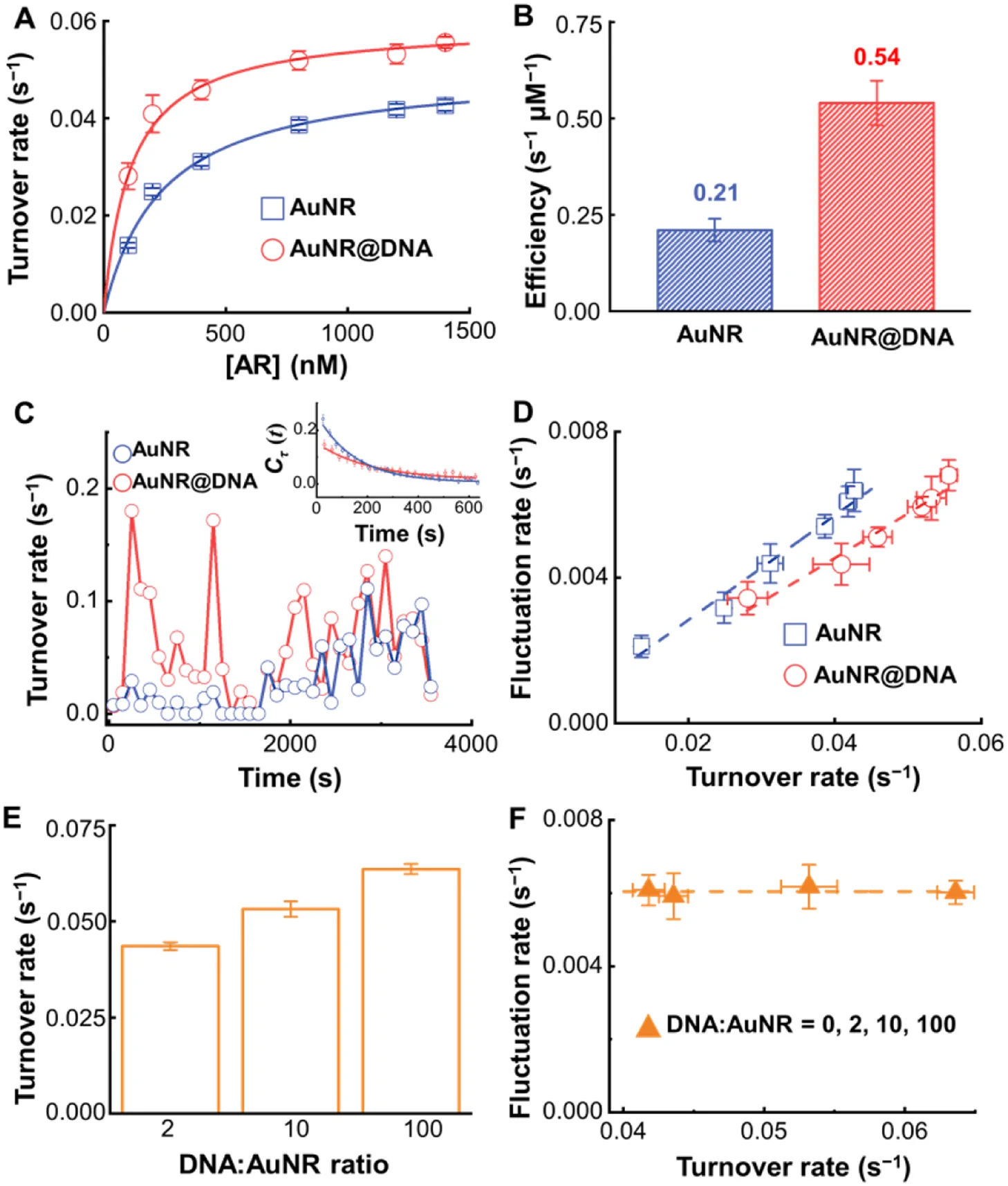
(A) Dependence of the single-particle turnover rate ⟨τ⟩^–1^ on AR concentration for bare AuNRs and AuNR@DNA. Solid lines are fits to eq 1 (B) Catalytic efficiency (*V*_max_/K_M_) for bare AuNR and AuNR@DNA. (C) Time-dependent turnover rate from a single AuNR (blue) and a single AuNR@DNA (red) at [AR] = 800 nM; rates calculated in 100 s windows. Inset: autocorrelation function C_τ_(t) fit to a single exponential decay (Supporting Information, Section S7.4). (D) Dependence of reactivity fluctuation rate on mean turnover rate for AuNR and AuNR@DNA; linear fits reflect adsorbate–surface interactions. (E) Dependence of turnover rate on DNA:AuNR feed ratio. [AR] = 800 nM. (F) Dependence of reactivity fluctuation rate on DNA:AuNR feed ratio. Dashed line is an empirical fit. All experiments: 50 mM sodium phosphate buffer (pH 7.4), 25 °C, [H_2_O_2_] = 60 mM. Error bars are s.e.m.; each data point averaged from >60 particles.

Coating the AuNR with a DNA corona to form AuNR@DNA substantially improved both kinetic parameters (Figure 4A, red). The maximum reactivity *V*_max_ increased to 0.059 ± 0.001 s^–1^, while *K*_M_ decreased from 0.24 ± 0.02 to 0.11 ± 0.01 μM, reflecting both additional reactive sites within the DNA corona and the stronger affinity of AR for DNA bases relative to the bare gold surface.^33^ Changes in substrate accessibility upon DNA functionalization are reflected in the decreased *K*_M_ of AuNR@DNA relative to bare AuNR and are accounted for in the Michaelis–Menten kinetic analysis; they do not confound the CID line width analysis, which is insensitive to substrate concentration under the conditions used for SPSS measurements. Together, these changes raised the catalytic efficiency to 0.54 ± 0.06 s^–1^ μM^–1^, a 2.6-fold improvement over bare AuNR (Figure 4B). Compared to the AuNS@DNA coronazyme reported previously (0.75 ± 0.03 s^–1^ μM^–1^),^33^ the AuNR@DNA efficiency is modestly lower, which we attribute to incomplete DNA coverage: the poly­(dA)_21_ containing hairpin (see Supporting Information S2 for structure and sequence), sized to coat the 5 nm AuNS in its entirety, covers only the tip regions of the 18 × 52 nm AuNR, leaving exposed gold sidewalls that catalyze AR oxidative conversion less efficiently than the DNA corona. This interpretation is supported by the reactivity fluctuation analysis described below.

The reactivity of individual AuNR@DNA particles fluctuated over time, a hallmark of dynamic disorder reflecting surface restructuring of the nanoparticle during catalysis^43,44^ (Figure 4C). Autocorrelation analysis of the microscopic waiting time series τ­(*t*) revealed that the fluctuation rate for bare AuNRs increases linearly with the mean turnover rate (Figure 4D), indicating substrate-assisted surface restructuring, the same adsorbate-surface interaction mechanism previously observed in AuNS.^33^ This linear dependence persists in AuNR@DNA, in contrast to the turnover-rate-independent fluctuations seen in the fully covered AuNS@DNA. This behavior confirms that exposed AuNR surfaces contribute to the measured catalytic signal even at DNA:AuNR feed ratios as high as 100:1, consistent with selective DNA binding at the rod ends due to the small poly­(dA)_21_ loop size.

To isolate the contribution of DNA coverage to catalytic performance, we varied the DNA:AuNR feed ratio from 2:1 to 10:1 to 100:1 during synthesis and measured *V*_max_ at a fixed AR concentration (Figure 4E). The maximum reactivity increased monotonically with DNA feed ratio, providing direct evidence that the DNA corona is responsible for the catalytic enhancement. The reactivity fluctuation rate remained indistinguishable across all DNA:AuNR feed ratios (Figure 4F), suggesting that exposed sidewalls dominate the fluctuation dynamics even at high DNA feed ratio. Full sidewall coverage would be expected to further improve both *V*_max_ and reaction selectivity and represent a clear target for future structural optimization. The persistence of turnover-rate-dependent reactivity fluctuations in AuNR@DNA across all DNA:AuNR feed ratios, combined with the feed ratio independence of the fluctuation rate (Figure 4F) and SYBR Gold quantification of bound DNA per particle (Figure S12 in the Supporting Information), collectively indicate that the poly­(A)-DNA hairpin preferentially occupies the high-curvature end facets of the AuNR, leaving the sidewall surface partially exposed. This geometric constraint, rather than the total DNA:AuNR feed ratio, governs the extent of DNA corona formation on the AuNR and accounts for the lower catalytic efficiency of AuNR@DNA relative to the fully covered AuNS@DNA coronazyme.

### CID at the AuNR-DNA Interface Drives Plasmon-Enhanced Catalysis

Having established the dark-state catalytic performance of the AuNR@DNA system, we addressed the central question of this work: can the longitudinal LSPR of the AuNR be harnessed to enhance catalytic activity, and if so, through what mechanism? Bare AuNRs showed no detectable change in turnover rate upon 680 nm illumination at any tested light intensity (Figure 5A, blue bars; Figure S7). This negative result is consistent with the well-established limitation of plasmonic photocatalysis: without a suitable molecular acceptor at the nanoparticle surface, hot carriers recombine within approximately 100 fs, far too rapidly to contribute to catalytic events.^35^ The citrate capping layer of bare AuNRs does not provide an effective charge-transfer interface, and the deposited plasmon energy dissipates entirely as heat. The increase in fluctuation rate with substrate concentration confirms that the exposed AuNR sidewall surfaces remain catalytically active through adsorbate-driven surface restructuring, indicating that catalytic turnover in AuNR@DNA is not exclusively confined to the DNA corona. In contrast, the plasmon-enhanced catalytic increment and its linear dependence on LED power density are observed exclusively in AuNR@DNA and not in bare AuNRs, supporting a photon-dependent, DNA-mediated interfacial charge transfer mechanism for the light-driven enhancement.

**Figure 5:**
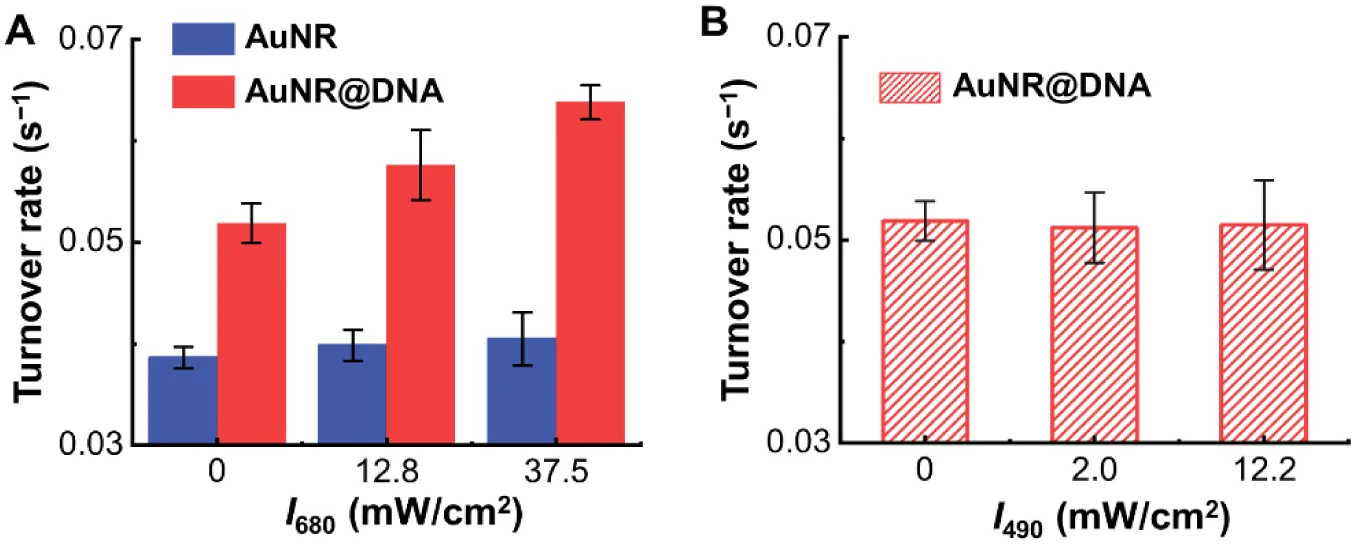
(A) Turnover rates for bare AuNR (blue) and AuNR@DNA (red) as a function of 680 nm LED power density. (B) Turnover rates for AuNR@DNA under 490 nm LED illumination. [AR] = 800 nM for all experiments. Error bars are s.e.m.; each data point averaged from >60 particles.

The AuNR@DNA system showed a clear, intensity-proportional increase in turnover rate upon 680 nm excitation (Figure 5A, red bars). The linear dependence on light intensity identifies a single-photon mechanism, and the selectivity for 680 nm, which excites the longitudinal LSPR while leaving interband transitions unperturbed,^35^ provides strong evidence that the observed enhancement is plasmon-driven rather than thermal. To rigorously exclude photothermal contributions, we performed a control experiment using 490 nm excitation, which deposits comparable thermal energy without activating the longitudinal LSPR.^7,35^ No reactivity enhancement was observed at any 490 nm power density (Figure 5B), conclusively establishing that interfacial charge transfer associated with CID, rather than local heating, is responsible for the catalytic improvement. Additionally, our calculation (Figure S13 and Table S3 in the Supporting Information) showed that the temperature increase due to the LED illumination is in the order of tens of microkelvin range, which is not enough to thermally drive the catalytic reaction by 20% as observed. Therefore, the enhanced photocatalysis can be attributed to plasmon enhancement.

It is important to distinguish CID from hot carrier injection, as these are related but mechanistically distinct processes. CID refers to the direct decay of the plasmon into an interfacial charge-separated state at the Au-DNA interface within ten femtoseconds, quantified here through LSPR line width broadening. Hot carrier injection refers to the subsequent, indirect process in which intraband hot carriers generated after plasmon dephasing diffuse to the nanoparticle surface and inject into the adsorbate. While plasmon excitation can induce interfacial charge transfer via CID and hot-carrier media pathways, we could not distinguish between them in our studies. Between these ultrafast events and the microsecond-to-millisecond time scale of catalytic turnover lie multiple competing processes, including carrier relaxation, electron-hole recombination, reverse charge transfer, and trapping, which are not directly resolved by our experimental platform. Only carriers that escape these competing pathways ultimately contribute to the charge transduction chain and drive catalytic turnover. The strong and systematic correlation between η_CT_ and the plasmon-enhanced catalytic rate across all DNA:AuNR feed ratios is consistent with this mechanistic picture, though we acknowledge that complete resolution of the intermediate carrier dynamics would require ultrafast time-resolved spectroscopy beyond the scope of the current study.

The central finding of this work emerges from comparing η_CT_ and the catalytic reactivity data across the same DNA:AuNR feed ratios. Both quantities increase in concert as DNA:AuNR feed ratio increases (Figures 2F and 5A), establishing the first direct, particle-level quantitative correlation between interfacial charge transfer efficiency and catalytic activity in a plasmonic system. This correlation is the experimental signature of the charge transduction chain: plasmon excitation induces electron transfer across the Au-DNA interface into the DNA corona via CID; charge transport along π-stacked DNA bases^45^ carries the electrons to substrate-bound reactive sites; and AR oxidation completes the catalytic cycle, restoring the DNA bases to their ground state (Figure 6). The plasmon excitations enhance interfacial charge transfer at the Au-DNA interface via CID, accelerating the generation and long-range transport of DNA radical cations that directly drive AR oxidative conversion within the corona phase; since H_2_O_2_ is present in large excess, interfacial charge injection and migration are proposed as the rate-limiting steps that govern the plasmon-enhanced catalytic activity. Each step in this chain is supported by independent experimental evidence, with CID confirmed by SPSS, corona-phase catalysis by SPFM, and DNA-mediated charge transport supported by prior plasmonic DNA-wire studies.^34^ The spatial separation between the site of charge generation at the AuNR surface and the catalytic turnover sites within the DNA corona constitutes the long-range character of the reaction, consistent with the well-established capacity of π-stacked DNA to mediate charge transport over nanometer-scale distances.^34,45^

**Figure 6:**
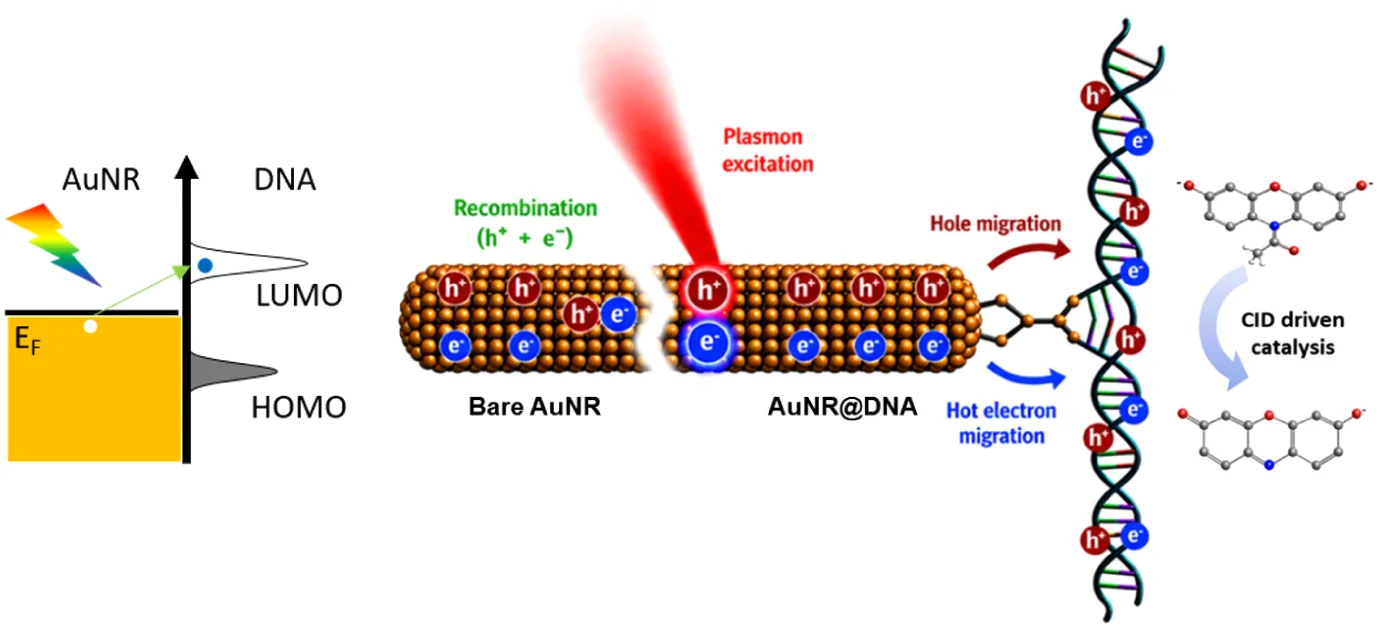
Mechanism of CID driven catalysis occurring at AuNR@DNA.

## Conclusions

The central challenge in plasmonic photocatalysis is not hot carrier generation but hot carrier survival: carriers are produced on femtosecond time scales yet must drive reactions that unfold over microseconds or longer. This work demonstrates that a programmable DNA corona resolves this mismatch by serving simultaneously as a CID-active charge-extraction interface and a long-range charge-transport medium that delivers those carriers to catalytically active sites nanometers away from the nanoparticle surface. By combining three single-particle techniques on the same system, we move beyond ensemble-averaged descriptions and reveal a direct, quantitative structure-function relationship that would be entirely hidden in bulk measurements: as DNA:AuNR feed ratio increases, both the CID efficiency and the plasmon-enhanced catalytic rate increase proportionally, at the level of individual nanoparticles.

The mechanistic picture that emerges is of a modular charge transduction chain: plasmon decays into charge separation states via CID where electrons and holes populate at DNA corona; π-stacking along the DNA backbone conducts the charges to substrate-bound reactive sites; and catalytic turnover is completed remotely, without direct contact with the metal surface. Each module in this chain is independently identifiable in our data, a degree of mechanistic resolution that single-particle methods uniquely afford. The agreement between the CID efficiency measured by SPSS and the catalytic enhancement measured by SPFM across three DNA:AuNR feed ratios provides, to our knowledge, the first quantitative, sample-level validation that improving interfacial charge transfer translates directly and predictably into faster catalysis. We note that while the quantitative correlation between η_CT_ and plasmon-enhanced catalytic rate provides strong experimental support for the proposed charge transduction pathway, our measurements report on the plasmon dephasing time scale and do not directly resolve the subsequent carrier dynamics between the femtosecond CID step and the microsecond catalytic turnover; complete mechanistic deconvolution of direct and indirect charge transfer pathways would require ultrafast time-resolved spectroscopy and represents an important direction for future work.

These findings carry implications that extend well beyond the specific AuNR@DNA system studied here. For the plasmonic catalysis community, they establish CID engineering as a quantitatively tractable handle for improving photocatalytic performance: rather than optimizing nanoparticle geometry or excitation wavelength alone, one can tune the molecular adsorbate layer to increase η_CT_ and thereby directly increase reaction rates. For the DNA nanotechnology community, the results demonstrate that the charge-transport properties of DNA, well established in purely electronic contexts, are functionally relevant in a hybrid plasmonic-catalytic system operating in aqueous solution under ambient conditions. For the broader nanoscience community, the coronazyme architecture illustrates how separating the functions of charge generation, charge transport, and substrate binding into distinct, independently optimizable structural domains can overcome fundamental limitations of single-material catalysts.

The 20% plasmon-driven catalytic enhancement observed here represents a conservative lower boundary: current AuNR@DNA geometry leaves substantial gold sidewall area uncovered, contributing a less efficient bare-surface catalytic pathway that dilutes the measured CID effect. We note that the plasmon-enhanced catalytic rate was characterized at a representative DNA:AuNR feed ratio (10:1); systematic measurements across all feed ratios to generate a direct correlation plot of plasmon-induced rate enhancement versus η_CT_ represent an important direction for future work that would further strengthen the quantitative structure-function relationship established here. Full sidewall DNA coverage, achievable through sequence engineering or multivalent hairpin designs, would be expected to amplify both the enhancement magnitude and the reaction selectivity. More broadly, the modular logic of the coronazyme framework^33,46,47^ invites systematic variation of both the nanocore (topology, composition, and resonance wavelength) and the corona (sequence, secondary structure, and charge-transport length) as orthogonal design parameters. Such a framework, now grounded in a quantitative CID-to-catalysis structure-function relationship, provides a rational path toward light-driven artificial enzymes with efficiencies surpassing those of natural metalloenzymes.

## Experimental Section

### Synthesis of AuNR@DNA Coronazymes

Oligonucleotides were purchased from Integrated DNA Technologies (IDT), NJ, USA and purified by standard polyacrylamide gel electrophoresis (PAGE). After purification, the oligonucleotides were stored in 1 mM Tris buffer (pH 7.4) at −20 °C until use. The sequences of all oligonucleotides are listed in Supporting Information Table S1. A detailed procedure for preparing the DNA construct that binds AuNR is provided in Supporting Information S2. Briefly, the synthesis involved three main steps: (1) phosphorylation of oligonucleotides ppd5.1 to ppd5.2, (2) annealing of oligonucleotide pairs ppd5.4/ppd5.5 and ppd5.2/ppd5.3, and (3) one-pot ligation of ppd5.1, the ppd5.2/ppd5.3 duplex, and the ppd5.4/ppd5.5 duplex. The final DNA hairpin product was purified by PAGE, verified, and stored in 1 mM Tris buffer (pH 7.4) until further use.

For preparation of the AuNR@DNA coronazyme, the purified DNA hairpin was mixed with citrate-capped AuNR colloidal solution at various DNA-to-AuNR feed ratios. The mixture was incubated at −80 °C for approximately 15 min to promote ligand exchange, during which citrate ligands on the AuNR surface were replaced by adenine residues in the poly­(dA) region of the DNA hairpin. The sample was then rapidly thawed to room temperature to redisperse the particles uniformly in solution.

The formation of AuNR@DNA complexes was characterized by ultraviolet-visible (UV-Vis) spectroscopy and SMFS, as shown in Supporting Information Figure S2. The DNA corona bound to the AuNR surface improved colloidal stability, prevented nanoparticle aggregation, and enabled stable suspension of the DNA/AuNR coronazymes in solution.

### SMFS Experiments and Data Analysis

The interaction between AuNR and the DNA construct was investigated using a dual-trap optical tweezers instrument. The optical tweezers instrument setup consisted of the DNA hairpin construct which was ligated to two double-stranded DNA (dsDNA) handles: a 1558-bp handle on the left side and a 2391-bp handle on the right side (see Supporting Information for synthesis). The 1558-bp dsDNA handle was functionalized with biotin at its distal end, whereas the 2391-bp dsDNA handle was functionalized with digoxigenin. These two labels enabled tethering of the construct between a pair of optically trapped beads through biotin–streptavidin and digoxigenin–anti-digoxigenin affinity interactions, as illustrated in Supporting Information S4. Accordingly, one bead was coated with streptavidin and the other with anti-digoxigenin antibody to facilitate formation of the tethered construct.

A three-channel microfluidic chamber was used to examine the interaction of the DNA hairpin with AuNR, as shown in Supporting Information S4. Anti-digoxigenin antibody-coated beads were introduced through one outer channel, while streptavidin-coated beads carrying the DNA construct were introduced through the opposite outer channel. These beads were then guided into the central channel through microcapillary connections. In the central channel, each bead was captured in a separate optical trap. A single tether was formed by bringing the two beads into close proximity, allowing the digoxigenin on the DNA construct to bind to the anti-digoxigenin antibody on the opposing bead.

During the SMFS experiment, force was applied to the tethered construct by moving the two optically trapped beads apart with a steerable mirror controlling one of the traps. The applied tension was determined from the trap stiffness and the displacement of each bead from the center of its trap. Force–extension (FX) data were collected using a LabVIEW program (National Instruments, Austin, TX) at a sampling frequency of 1 kHz and a loading rate of 5.5 pN/s over the 10–30 pN force range. The raw data were processed using custom-written MATLAB scripts (The MathWorks) and subsequently analyzed in IGOR Pro v6.37 (WaveMetrics). Custom IGOR procedures were then used to generate the final force–extension curves.

### SPSS Experiments and Data Analysis

Detailed procedures of single-particle scattering measurements and their data analysis can be found in Supporting Information S5. Essentially, AuNR@DNA were spin-casted on a coverslip, which was assembled into a flow cell with another glass slide spaced by a parafilm. The flow cell was mounted on a custom-built dark-field scattering microscope with a hyperspectral capability. A PBS solution with a pH of 7.4 flowed into the cell, and then the hyperspectral images of the sample were acquired. The scattering spectra of individual AuNRs were background-subtracted and normalized by the spectrum of the lamp. A custom-written MATLAB program was employed to obtain single-particle scattering spectra from the hyperspectral images. The spectrum was fit to a Lorentzian function to get the peak energy (*E*_res_), full width at half maximum (Γ), and peak intensity (*I*_max_).

### SPFM Experiments and Data Analysis

Detailed procedures of single-particle catalysis and data analysis are described in Supporting Information Sections S6 and S7. Briefly, bare AuNRs or AuNR@DNA were sparsely dispersed on a clean coverslip, which was then assembled into a microfluidic reaction chamber with microscope slide, double-sided tape and epoxy glue. This chamber was connected to a syringe pump at a 20 μL min^–1^ steady flow of reaction mixture. During the measurements, this microreactor was placed under a fluorescence microscope configured in Total Internal Reflection (TIR) mode (Figure S9). The reaction mixture is a 50 mM pH 7.4 phosphate buffer containing 0.2–1.6 μM Amplex Red (AR) and 60 mM H_2_O_2_. A 532 nm continuous wave laser beam (DragonLaser) of ∼8 mW was focused onto an area of ∼35 × 35 μm^2^ to excite the fluorescent emission of the product resorufin (RF).

To generate the LSPR of AuNRs, we mounted a 490 nm/680 nm LED light (Thorlabs Inc.) in the transmitted illuminator. The power density at the reactor’s surface was adjusted from 0 to 40 mW cm^–2^). A 100× N.A. = 1.49 oil immersion objective (UAPON 100×OTIRF, Olympus) collects the fluorescence signals for fluorogenic reaction. The signals are subsequently filtered by a longpass (Chroma, ET542lp) and a bandpass (Chroma ET575/50m) filter before reaching an sCMOS camera (Photometrics Prime 95B). We used the Olympus CellSens Dimension software to control the camera to operate at a frame rate of 50 ms. Lastly, the recorded movies were analyzed using ThunderSTORM,^48^ an ImageJ-based program, in combination with a home-written MATLAB program to extract the kinetic information for coronazyme catalysis (Supporting Information Section 7.2).

## Supplementary Material



## Notes

DNA oligonucleotide sequences and synthesis protocol for the poly­(A)-DNA hairpin construct; UV-Vis characterization and transmission electron microscopy of AuNR@DNA coronazymes including aspect ratio distributions; optical tweezers setup, microfluidic chamber schematic, and theoretical contour-length calculations for single-particle force spectroscopy; sample preparation procedure and hyperspectral dark-field microscopy setup for single-particle scattering spectroscopy; Lorentzian fitting procedure and derivation of the charge transfer efficiency η_CT_; fluorogenic probing reaction scheme and long-range catalytic mechanism for AuNR@DNA coronazyme-catalyzed Amplex Red oxidative conversion; single-particle fluorescence microscopy instrumentation, data processing pipeline including two-dimensional Gaussian localization, modified Michaelis–Menten kinetic parameters for AuNR, AuNR@DNA, AuNS, and AuNS@DNA, reactivity fluctuation analysis under dark and illuminated conditions, and plasmon-enhanced catalysis experimental protocol (PDF)
