# Supplementary material for: Interfacial Charge Transfer Controls Plasmon-Enhanced Catalysis in DNA-Coated Gold Nanorods
Source: ACS Nano. 2026 Sep 18;20(38):26074–84. doi: 10.1021/acsnano.6c08886 (PMC13629681; doi:10.1021/acsnano.6c08886)
Supplement: Supplementary file 1 [file nn6c08886_si_001.pdf]

Supporting Information

Interfacial Charge Transfer Controls Plasmon-Enhanced Catalysis in DNA-Coated Gold Nanorods

Bishal Pokhrel[†, 1], Li Zuo[†, 1], Tamie Vo[†, 3], Pravin Pokhrel[1], Rebecca L. Stratton[1], Sajan Shakya[1], Adeola Adeyemi[1], Hanbin Mao*[, 1, 2], Wei-Shun Chang*[, 3], Hao Shen*[, 1, 2]

[1]Department of Chemistry and Biochemistry, Kent State University, Kent, Ohio, 44242, USA

[2]Advanced Materials and Liquid Crystal Institute, Kent State University, Kent, Ohio, 44242, USA

[3]Department of Chemistry and Biochemistry, University of Massachusetts, Dartmouth, Dartmouth, Massachusetts, 02747, USA

[†]These authors contributed equally

*Corresponding authors

Contents

S1. Materials 2
S2. Synthesis of the Poly(A)-DNA Hairpin 2
S3. Synthesis and Characterization of AuNR@DNA 3
S3.1 Preparation of AuNR@DNA via the Freeze-Thaw Method 3
S3.2 Transmission Electron Microscopy of AuNRs and AuNR@DNA 4
S4. Optical Tweezers Setup and Single-Particle Force Spectroscopy 4
S5. Single-Particle Scattering Spectroscopy of AuNR@DNA 6
S5.1 Sample Preparation 6
S5.2 Hyperspectral Dark-Field Microscopy Setup 6
S5.3 Lorentzian Fitting and Charge Transfer Efficiency Calculation 7
S5.4 Uncertainty Propagation for $\boldsymbol{\Gamma}$**CID** and $\boldsymbol{\eta CT}$ 9
S5.5. Effect of Refractive Index Change on the Plasmon Linewidth 10
S6. Fluorogenic Probing Reaction and Long-Range Catalytic Mechanism 11
S7. Single-Particle Fluorescence Microscopy: Instrumentation and Data Analysis 12
S7.1 Instrumentation 12
S7.2 Single-Molecule Data Processing 13
S7.3 Modified Michaelis-Menten Kinetics 14
S7.4 Reactivity Fluctuation Analysis 16
S7.5 Plasmon-Enhanced Coronazyme Catalysis: Experimental Protocol 17
S7.6 SYBR Gold fluorescence quantification of DNA content on AuNRs 18
S8. Estimation of temperature rise on AuNR under photoexcitation 19

Supporting Information References 21

S1. Materials

Citrate-capped gold nanorods (AuNRs, 18 × 52 nm) were purchased from Nanopartz™ (Loveland, CO). All oligonucleotides were purchased from Integrated DNA Technologies (IDT, Coralville, IA) and further purified by polyacrylamide gel electrophoresis followed by ethanol precipitation. All enzymes were purchased from New England Biolabs (NEB Inc., Ipswich, MA). Hydrogen peroxide solution (30% w/w) was purchased from Spectrum Chemical Corp. Amplex Red (AR, ≥95% purity) and SYBR™ Gold Nucleic Acid Gel Stain were purchased from Invitrogen. Unless otherwise noted, all reagents were used as received without further purification.

S2. Synthesis of the Poly(A)-DNA Hairpin

Table S1. List of DNA oligonucleotides

Oligo ID	# nts	Sequence (5' – 3')
ppd 5.1	24	GTC CGG ACC CTG TTTT CAG GGT CC
ppd 5.2	48	CTG TGA GGT AGT AGA AAA AAA AAA AAA AAA AAA AAC TTC AGT CTA GCG
ppd 5.3	48	GGA CCG CTA GAC TGA AG AAA AAA AAA AAA AAA AAA AAA CTA CTA CCT C
ppd 5.4	39	CGG TAC GGT GTC AAA TAC CGC ACA GAT GCG ACG GTC GAT
ppd 5.5	39	ACAG ATC GAC CGT GCC AGC AAG ACG TAG CCC AGC GCG TC
BsaI HF	30	CAG GGA CGC GCT GGG CTA CGT CTT GCT GGC
Xbai	26	CGC ATC TGT GCG GTC TTT CAC ACC GT

Five oligos (see Table S1 for sequence) were separately phosphorylated. Up to 500 pmol of DNA 5′ termini were phosphorylated in a 50 µl reaction containing 5 µl of 10X T4 DNA ligase buffer containing 1 mM ATP, 1 µl of T4 polynucleotide kinase (10 units), and nuclease-free water to volume. The reaction mixture was prepared in a microcentrifuge tube on ice, then incubated at 37°C for 3 hours. Following incubation, the enzyme was heat-inactivated by incubating the reaction at 65°C for 20 minutes. Following phosphorylation, phosphorylated oligo pair ppd5.2/5.3 and ppd5.4/5.5 were separately annealed to hybridize. Annealing was done by heating the mixture 1:1 mol ratio of each pair at 95°C for 10 minutes and slowly cooling down to 16°C at the rate of -1°C/minute. Finally, the two pairs and the oligo ppd5.1 were ligated to covalently link each fragment to form the final DNA hairpin construct. A ligation reaction was assembled in a microcentrifuge tube on ice in a total volume of 20 µl. The reaction contained 2 µl of 10X T4 DNA

ligase buffer, 1 µM each of ppd5.1, ppd5.2/5.3 and ppd5.4/5.5 annealed oligo pair, nuclease-free water to volume, and 1 µl of T4 DNA ligase (40U/µL) added last. The reaction was gently mixed by pipetting up and down and briefly microcentrifuged. The reaction was incubated at 16°C overnight. Following ligation, the reaction was heat-inactivated at 65°C for 10 minutes.

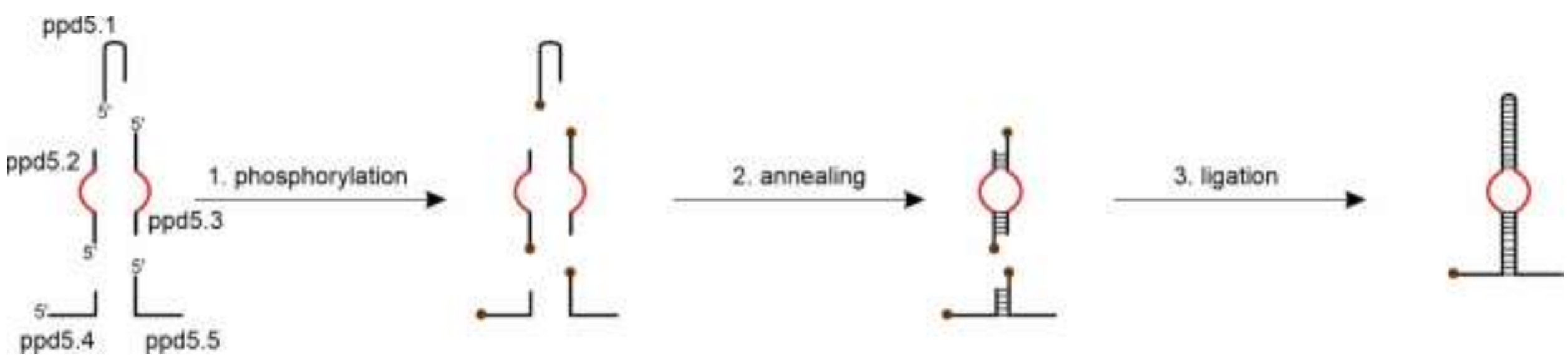

Figure S1. Synthesis of the DNA hairpin construct for single molecule catalysis experiment.

S3. Synthesis and Characterization of AuNR@DNA

S3.1 Preparation of AuNR@DNA via the Freeze-Thaw Method

AuNR@DNA coronazymes were prepared using a freeze-thaw ligand exchange procedure. Briefly, citrate-capped AuNR colloidal solution (18 × 52 nm) was mixed with the poly(A)-DNA hairpin at the desired feed ratio (DNA: AuNR = 2:1, 10:1, 100:1, or 200:1). The mixture was stored at −80 °C for approximately 20 min, during which a ligand exchange occurred at the AuNR surface as citrate was displaced by the poly(adenosine) loop of the DNA hairpin. The mixture was then thawed at room temperature to homogenize the AuNR@DNA suspension prior to use. UV-Vis absorption spectra confirmed successful AuNR@DNA formation. Citrate-capped AuNRs exhibit characteristic transverse and longitudinal plasmon peaks at approximately 515 nm and 699 nm, respectively (**Fig. S2A**). Both peaks were preserved after the freeze-thaw procedure in the presence of poly(A)-DNA (**Fig. S2B**, with DNA), consistent with well-dispersed AuNR@DNA. The incorporation of DNA to the nanorods confirms that they do not aggregate, minimizing the potential of dimers and multimers formation.

In the absence of DNA, the longitudinal peak was strongly suppressed following the freeze-thaw process (**Fig. S2B**, without DNA), indicating irreversible aggregation of unprotected AuNRs. The retention of the plasmon peaks in the presence of DNA provides direct evidence of AuNR@DNA coronazyme formation and colloidal stability.

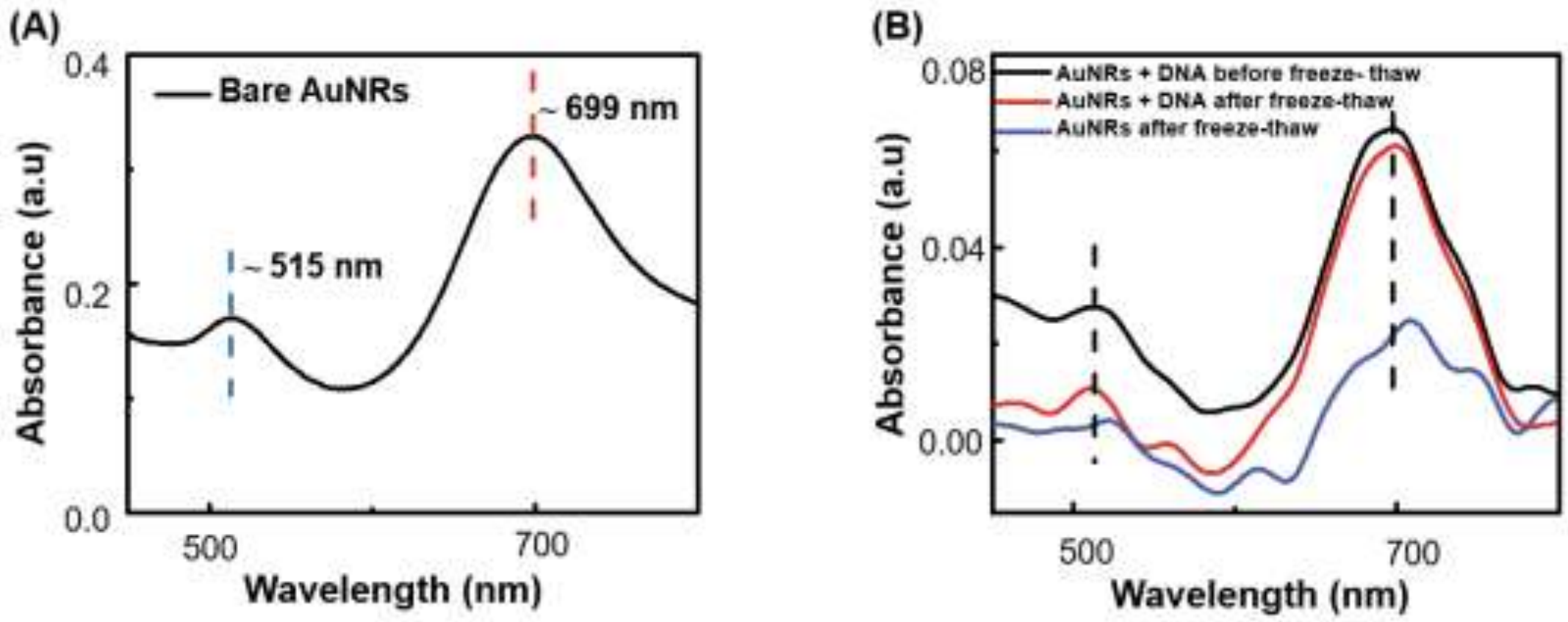

Figure S2. UV-Vis absorption spectra of (A) citrate-capped AuNRs and (B) AuNR suspensions after the freeze-thaw procedure in the presence (red) and absence (black) of poly(A)-DNA.

S3.2 Transmission Electron Microscopy of AuNRs and AuNR@DNA

The morphology of AuNRs and AuNR@DNA coronazymes was characterized by transmission electron microscopy (TEM; FEI Tecnai G2 F20). Because of the low electron density contrast of DNA relative to gold, the DNA corona is not directly visible in TEM images (**Fig. S3A**). Statistical analysis of 356 citrate-capped AuNRs and 569 AuNR@DNA particles yielded mean aspect ratios of 2.80 and 2.77, respectively (**Fig. S3B, C**), confirming that the freeze-thaw functionalization procedure does not alter the nanorod morphology.

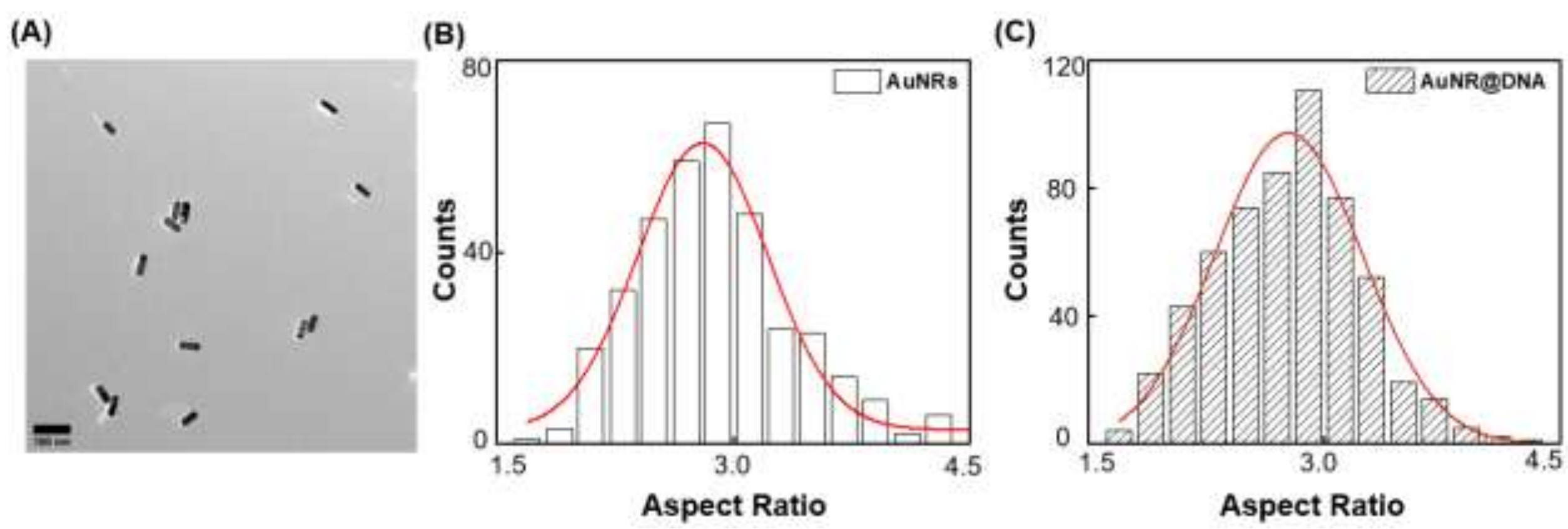

Figure S3. TEM Images of (A) AuNRs. Aspect ratio distributions for (B) citrate capped AuNRs and (C) AuNR@DNA coronazymes, determined from TEM images.

S4. Optical Tweezers Setup and Single-Particle Force Spectroscopy

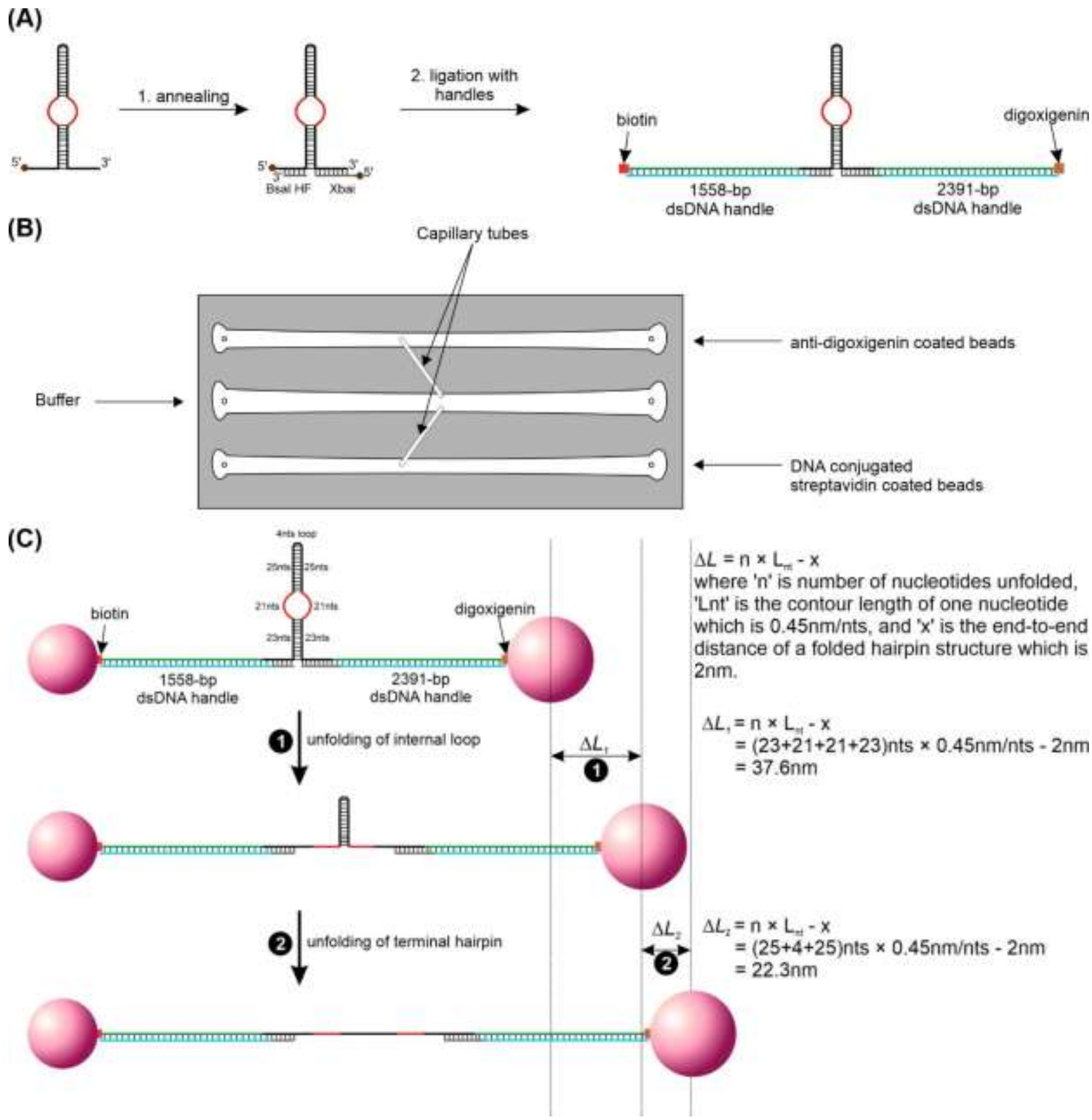

Figure S4. (A) Synthesis of a single-molecule DNA hairpin construct for optical tweezers experiment. (B) Schematic of a microfluidic chamber used in single-molecule force spectroscopy experiment. (C) Calculation of theoretical change-in-contour-length of the construct during its unfolding in the optical tweezers instrument.

The synthesized DNA hairpin construct synthesized was annealed with BsaI HF and Xbai in 1:1:1 mol ratio at a final concentration of 1 μM. Annealing was done by heating the mixture at 95°C for 10 minutes and slowly cooling down to 16°C at the rate of −1°C/minute. It was then followed by ligation with 1558-bp and 2391-bp dsDNA handles. A ligation reaction was assembled in a microcentrifuge tube on ice in a total volume of 10 µl. The reaction contained 1 µl of 10X T4 DNA ligase buffer, 0.1 μM each of 1558-bp and 2391-bp dsDNA handles and 0.5 μM of the DNA hairpin, nuclease-free water to volume, and 0.5 µl of T4 DNA ligase (40U/μL) added last. The reaction was gently mixed by pipetting up and down and briefly microcentrifuged. The reaction was incubated at 16°C overnight. Following ligation, the reaction was heat-inactivated at 65°C for 10 minutes. The biotin functional group in the 5' end of 1558-bp dsDNA handle and the digoxigenin in the 3' end of 2391-bp dsDNA handle allowed the construct to form a tether in between a pair of optically trapped beads via streptavidin/biotin and digoxigenin/antidigoxigenin affinity linkages.

S5. Single-Particle Scattering Spectroscopy of AuNR@DNA

S5.1 Sample Preparation

Glass slides with two pre-drilled holes (Fisher Scientific, 25 × 75 × 1 mm, 125444) and coverslips (Thermo Scientific, 24 mm × 60 mm No. 1.5, 3423) were cleaned by sequential sonication in Hellmanex (2%), reagent alcohol (Fisher Scientific, 89 to 91%, A995−4), acetone (Fisher Scientific, 99.5%, A929−4), potassium hydroxide (Fisher Scientific, KOH, 1 M, P250−1), and deionized water. The AuNR@DNA solution was drop-cast onto the cleaned coverslip, which was then assembled into a flow cell using a glass slide and parafilm as a spacer. The two tubes (Scientific Commodities, BB31695-PE/4) were connected to the pre-drilled holes via tubing connectors (Grace Bio-Labs, 460003) and sealed with epoxy.

S5.2 Hyperspectral Dark-Field Microscopy Setup

Single-particle DFS measurements were performed using a custom-built DFS microscope, which integrated an inverted microscope (Zeiss, Axio-Observer 5) and a spectrometer (Princeton Instruments, ARC-SP-2156) equipped with a CCD camera (Princeton Instruments, PIX-400BRX) mounted on a motorized linear stage (Newport, CONEX-LTA-HL). White light from a halogen lamp (Zeiss, HAL-100) was directed through a dark-field condenser (N.A. = 1.4) and focused on the sample. The scattered light from the AuNRs was collected by an oil immersion objective

(Zeiss, Plan-Achromat, N.A. = 0.7, 63×) and directed to the spectrometer located at the first image plane of the microscope. A custom-written LabVIEW program synchronized the motorized linear stage and the spectrometer, enabling hyperspectral imaging by scanning the spectrometer slit across the scattering image during simultaneous spectral acquisition. Analyses of the hyperspectral images were carried out by a custom-written MATLAB program developed in the lab. All reported scattering spectra were integrated with a 5 × 5 pixel area around the bright spot centroids, background-subtracted, and normalized by the lamp spectrum.

S5.3 Lorentzian Fitting and Charge Transfer Efficiency Calculation

The scattering spectrum of each individual AuNR or AuNR@DNA particle was fit to a Lorentzian function to extract the resonance peak energy (E_{res}), linewidth (Γ), and peak intensity (I_{max}). To exclude spectral contributions from interband transitions, only particles with $E_{res} < 1.78$ eV were included in the analysis.

The CID-attributed linewidth broadening was quantified using the charge transfer efficiency η_{CT}:

$$\eta_{CT} = \Gamma_{CT} / \Gamma \qquad \text{(S1)}$$

where $\Gamma_{CT} = \Gamma_{AuNR@DNA} - \bar{\Gamma}_{AuNR}$ is the linewidth increment of a given AuNR@DNA particle relative to the mean linewidth of as-received citrate-capped AuNRs ($\bar{\Gamma}_{AuNR} = 91 \pm 8$ meV), and Γ is the measured linewidth of that particle. η_{CT} thus represents the fraction of the total plasmon linewidth attributable to interfacial charge transfer to the adsorbed DNA corona. This approach is consistent with the treatment established by previous publications.[S1-5] Individual AuNRs were identified in the hyperspectral dark-field images by their characteristic single-peak Lorentzian scattering spectra. Particles exhibiting split plasmon peaks, non-Lorentzian lineshapes, or anomalously high scattering intensities, indicative of coupled dimers or aggregates, were excluded from the linewidth analysis. AuNRs were prepared at a surface density of ~0.1 particles μm^{-2} to minimize particle overlap in the diffraction-limited images. For the selected single AuNRs, the Γ was defined as the full width at half maximum (FWHM) extracted from the best-fit parameters within a fixed spectral window, with only high-quality Lorentzian fits ($R^2 \geq 0.95$) included in the statistical analysis. Examination of the fitting residuals ($Y_{exp} - Y_{fit}$) showed points tightly and randomly distributed around zero across the spectral range, confirming that the single-peak

Lorentzian model accurately describes the measured scattering spectra and does not introduce significant fitting artifacts into the extracted linewidths (Figure S5).

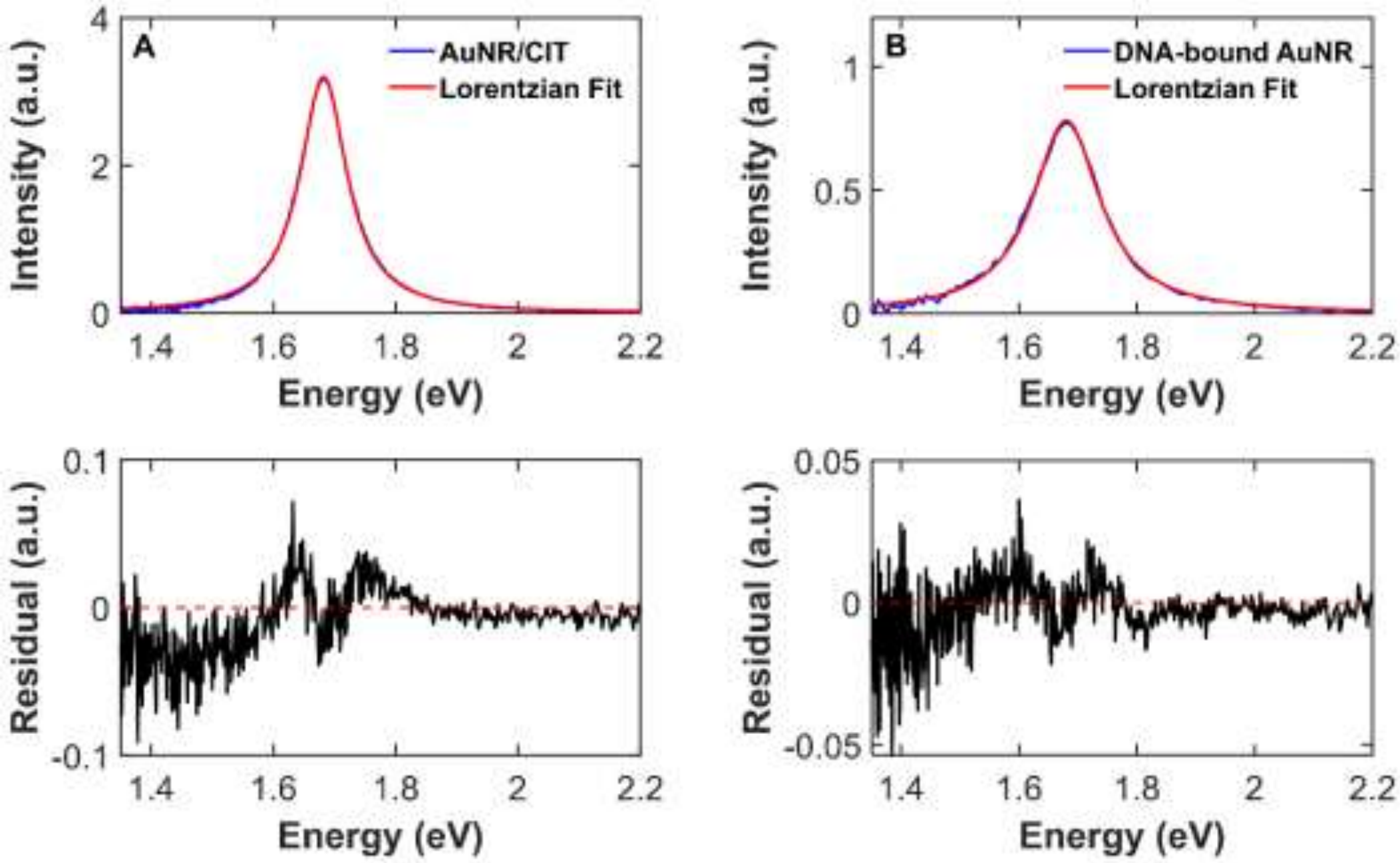

Figure S5. Representative Lorentzian fits with corresponding residuals for a bare AuNR/CIT (A) and AuNR@DNA (B).

By utilizing single-particle spectroscopy rather than ensemble measurements, ensemble inhomogeneous broadening arising from sample polydispersity is inherently eliminated. Furthermore, given small dimensions of the AuNRs (18 × 52 nm), radiative damping contributes only weakly to the total linewidth.[S1, 6] In addition, the physical dimensions of the nanorods remain unchanged upon functionalization, Γ_{rad} enters equally into both the bare AuNR baseline ($\bar{\Gamma}_{\mathrm{AuNR}}$) and the AuNR@DNA measurements. Subtracting this baseline ($\Gamma_{CT} = \Gamma_{\mathrm{AuNR@DNA}} - \bar{\Gamma}_{\mathrm{AuNR}}$) effectively isolates the CID contribution, allowing us to confidently attribute the systematic linewidth broadening to CID. In addition, due to intrinsic particle-to-particle size dispersion, individual particles on the smaller end of the size distribution may exhibit total linewidths below $\bar{\Gamma}_{\mathrm{AuNR}}$ ($\Gamma_{\mathrm{AuNR@DNA}} < \bar{\Gamma}_{\mathrm{AuNR/CIT}}$). These negative values represent physical statistical variance around the reference baseline. All negative data points were retained in the statistical calculations to avoid positive selection bias.

To rule out morphological artifacts, resonance energy (E_{res})-matched sub-analysis was performed. The results confirmed that the Γ broadening trend is not driven by geometric heterogeneity (**Fig. S5**). Across the E_{res} range of 1.35 - 1.76 eV, Γ and η_{CT} of AuNR@DNA exhibit only small variations without a clear trend. The standard deviations at a given E_{res} likely arise from intrinsic particle-to-particle size dispersion among the AuNRs. To explicitly determine whether η_{CT} depends on E_{res}, we averaged Γ and η_{CT} for single AuNRs within 0.5 eV energy bins. Figure S6 displays the average Γ (**A**) and η_{CT} (**B**) as a function of binned E_{res} for AuNR@DNA at different DNA: AuNR feed ratios, where the symbols and error bars represent the mean and standard deviation, respectively. Particles with $E_{res} < 1.5$ eV were grouped into a single bin due to the lower number of nanorods resonating at these lower energies. Overall, the binned average Γ and η_{CT} remain constant across the 1.35 to 1.76 eV range, confirming that the observed broadening is independent of E_{res}.

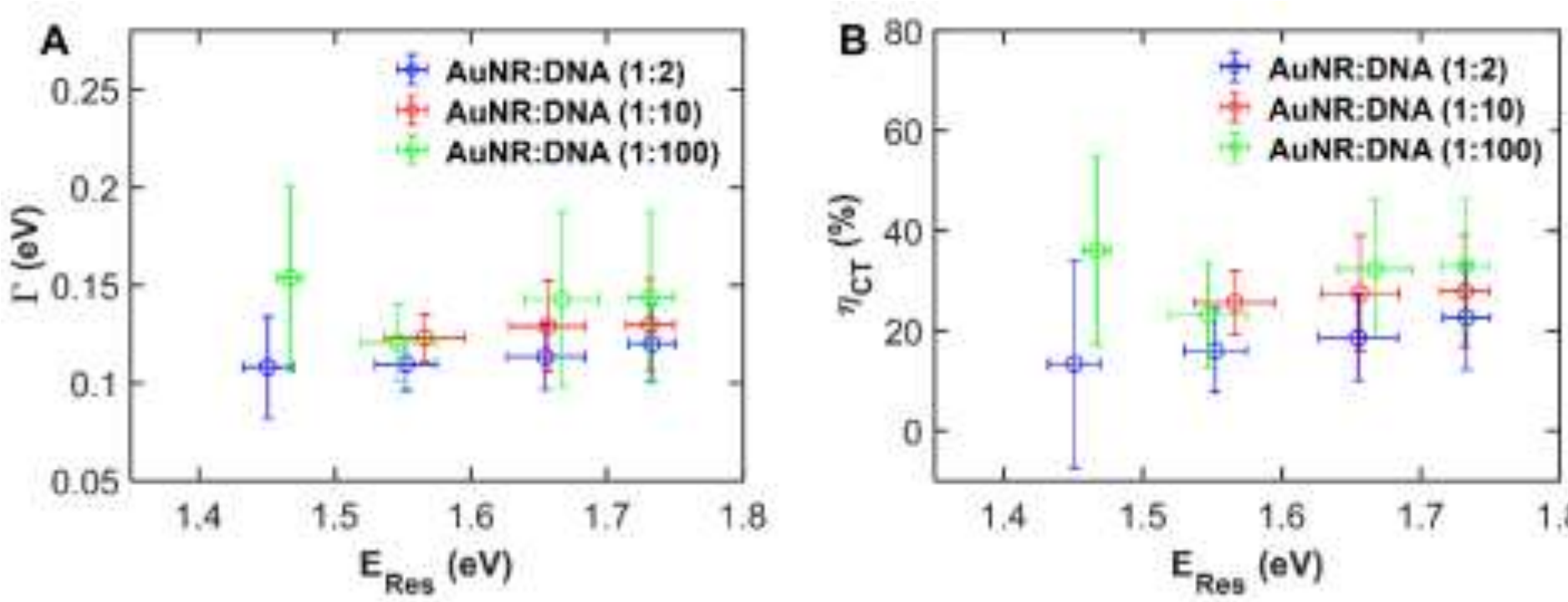

Figure S6. Average Γ and η_{CT} as a function of binned E_{res} for DNA: AuNR feed ratios of 2:1, 10:1, and 100:1. The symbols and error bars represent the mean and standard deviation of the values with bins of 0.5 eV in E_{res}.

S5.4 Uncertainty Propagation for $\boldsymbol{\Gamma}_{\mathbf{CID}}$ and $\boldsymbol{\eta}_{\boldsymbol{CT}}$

The electron transfer efficiency (η) for each nanoparticle NP_i

$$\eta_i = \frac{\Gamma_{\mathrm{AuNR@DNA},i} - \bar{\Gamma}_{\mathrm{AuNR/CIT}}}{\Gamma_{\mathrm{AuNR@DNA},i}} = 1 - \frac{\bar{\Gamma}_{\mathrm{AuNR/CIT}}}{\Gamma_{\mathrm{AuNR@DNA},i}} \quad \text{(S2)}$$

Thus, the uncertainty in each value is

$$\sigma_{\eta_i} = \frac{8\ meV}{\Gamma_{\text{AuNR@DNA},i}} \tag{S3}$$

where $\bar{\Gamma}_{\text{AuNR/CIT}} = 91 \pm 8$ meV

To report $\bar{\eta}_i \pm \sigma_{\bar{\eta}}$

$$\bar{\eta}_i = \frac{\sum_i \eta_i}{N_i} \tag{S4}$$

and the standard deviation was calculated as

$$\sigma_{\bar{\eta}} = \text{std}(\bar{\eta}_i) \tag{S5}$$

The uncertainty in the mean electron transfer efficiency was calculated as

$$SE.\sigma_{\bar{\eta}} = \sqrt{\sigma_{\bar{\eta}}^2 + \sum_i \frac{\sigma_{\eta_i}^2}{N_i}} \tag{S6}$$

where N_i is the number of AuNR@DNA particles in the corresponding dataset.

The resulting efficiencies are 20 ± 12%, 28 ± 13%, and 32 ± 15% for the DNA: AuNR feed ratios of 2:1, 10:1, and 100:1, respectively. These propagated uncertainties confirm that the observed increasing trend of CID contribution and electron transfer efficiency with increasing DNA loading remains distinct and well-defined.

S5.5. Effect of Refractive Index Change on the Plasmon Linewidth

To isolate the contribution of local refractive index changes from CID upon DNA functionalization, we calculated the scattering spectra of AuNRs coated with an adsorbed dielectric layer across a refractive index range of $n = 1.33$ (PBS buffer, no coating) to $n = 1.50$ (DNA coating, full coverage) shown in Figure S7A and S7B. These calculations were performed using the theoretical framework described by Foerster et al.[S1] The scattering cross section (C_{sca}) calculations reveal that local dielectric variations reduce the plasmon linewidth by less than 1 meV. This negligible variation cannot account for the significantly larger 25-51 meV broadening

observed experimentally upon DNA binding. We can therefore conclude that changes in the dielectric environment play a negligible role, and that CID is the primary mechanism driving the observed plasmon linewidth broadening upon DNA adsorption.

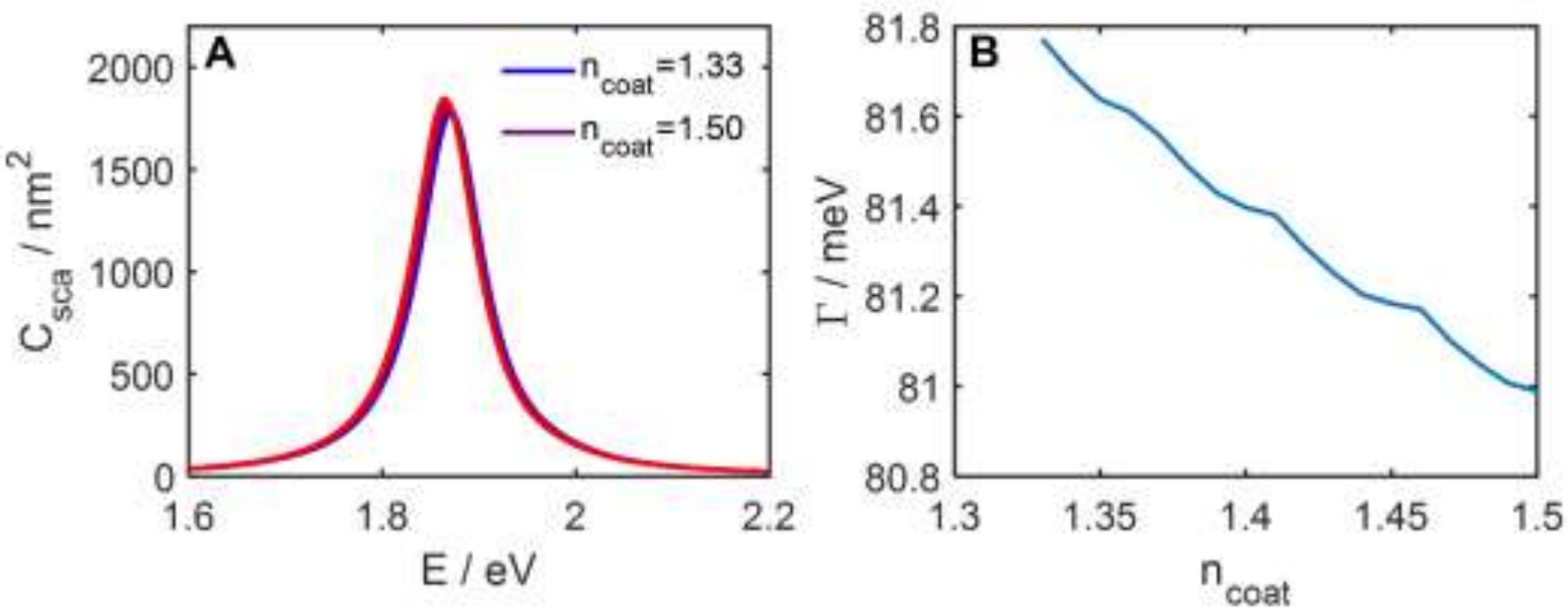

Figure S7. Effect of refractive index on plasmon linewidth. (A) Simulated scattering spectra and (B) corresponding linewidth (*Γ*) of AuNRs with an increasing coating refractive index (n_{coat}).

S6. Fluorogenic Probing Reaction and Long-Range Catalytic Mechanism

The oxidative conversion of amplex red (AR) to the highly fluorescent product resorufin (RF) was used as the fluorogenic probe reaction to evaluate the peroxidase-mimicking activity of AuNR@DNA coronazymes. Citrate-capped AuNRs catalyze AR oxidative conversion in the presence of H_2O_2, while the standalone poly(A)-DNA hairpin is catalytically inactive for this reaction. Upon DNA functionalization, the AuNR@DNA system exhibits significantly enhanced peroxidase activity through a long-range catalytic mechanism, as established in our prior work on AuNS@DNA coronazymes.[S7]

The proposed mechanism proceeds in three sequential steps (**Fig. S8**): (1) H_2O_2 undergoes homolytic cleavage at the AuNR surface, generating hydroxyl radicals (•OH) that oxidize adjacent DNA bases into radical cations; charge transfer then propagates along the π-stacked DNA backbone toward substrate-bound reactive sites. (2) AR molecules adsorbed at the DNA corona react with the arriving radical cations, converting AR to RF through two consecutive one-electron transfer steps. (3) RF desorbs from the DNA corona, regenerating the reactive site for the next catalytic cycle. Within this mechanism, CID under longitudinal LSPR excitation enhances the initial charge injection from the AuNR surface into the DNA corona, thereby accelerating all

downstream catalytic steps. As the H_2O_2 is present in large excess in the reaction mixture, the interfacial charge injection from the AuNR into the DNA corona and its subsequent long-range charge migration are the proposed rate-limiting step. The plasmon excitation increases the generation and transport of the hot charge species necessary for the AR oxidative conversion, resulting in enhanced catalysis.

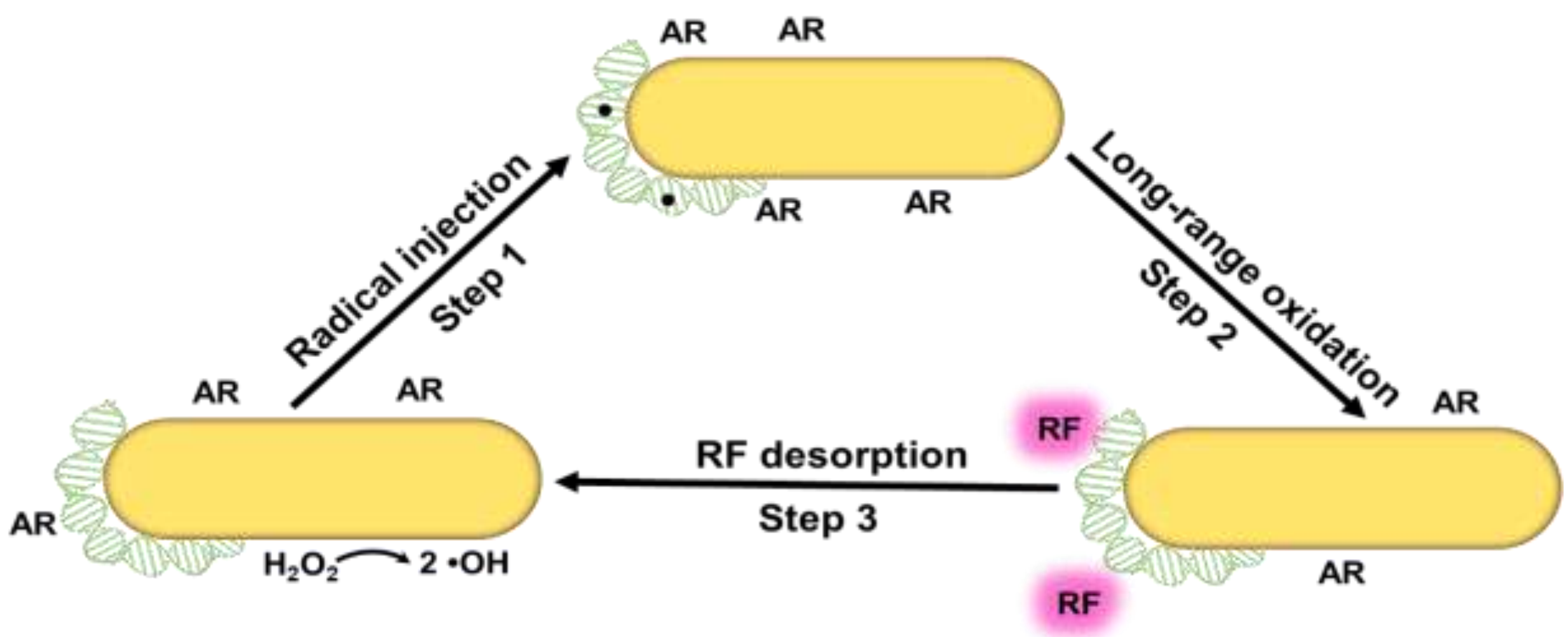

Figure S8. Schematic of the proposed long-range catalytic mechanism for AuNR@DNA coronazyme-catalyzed AR oxidative conversion. Step 1: radical generation and charge injection at the AuNR surface. Step 2: charge transport along the DNA backbone to the substrate-bound reactive site and AR-to-RF conversion. Step 3: RF desorption and site regeneration.

S7. Single-Particle Fluorescence Microscopy: Instrumentation and Data Analysis

S7.1 Instrumentation

Single-particle catalysis was performed inside a microfluidic chamber in which the nanoparticles (~1 nM) were immobilized on the coverslip surface and exposed to a continuous flow of reaction mixture. (**Fig. S9**). Briefly, 50 µL of approximately 10 pM citrate-capped AuNR or AuNR@DNA colloidal solution was drop-cast onto a clean glass coverslip (VWR, micro cover glass, No. 1, 25 × 25 mm) and allowed to adsorb for approximately 30 min. The coverslip was rinsed with MilliQ water for 3 min to remove weakly adsorbed particles, then assembled into a microfluidic reaction chamber (25 mm × 10 mm × 100 µm) using a microscope slide (Thermo

Scientific™), double-sided tape, and epoxy glue. Two holes drilled in the microscope slide served as inlet and outlet ports for polyethylene tubing.

The reaction chamber was connected to a syringe pump delivering a steady flow (20 µL min^{-1}) of reaction mixture: 0.2–1.6 µM AR and 60 mM H_2O_2 in 50 mM sodium phosphate buffer (pH 7.4). A 532 nm continuous-wave laser (~8 mW, DragonLaser) was focused onto a ~35 × 35 µm^2 area to excite the fluorescence of RF products. To excite the longitudinal LSPR of AuNRs, a 680 nm or 490 nm LED (Thorlabs Inc.) was mounted in the transmitted illumination path at power densities of 0–40 mW cm^{-2} (680 nm) or 0–15 mW cm^{-2} (490 nm). Fluorescence signals were collected through a 100× oil immersion objective (N.A. = 1.49, UAPON 100×OTIRF, Olympus), filtered by longpass (ET542lp, Chroma) and bandpass (ET575/50m, Chroma) filters, and recorded by an sCMOS camera (Photometrics Prime 95B) at 50 ms per frame using Olympus CellSens Dimension software.

Individual AuNR and AuNR@DNA particles were identified by their photoluminescence signals in the background frame. Particles with photoluminescence intensities substantially exceeding that of a single nanorod were excluded as likely dimers, multimers, or aggregates. The DNA corona provides additional colloidal stabilization that reduces nanorod aggregation during sample preparation.

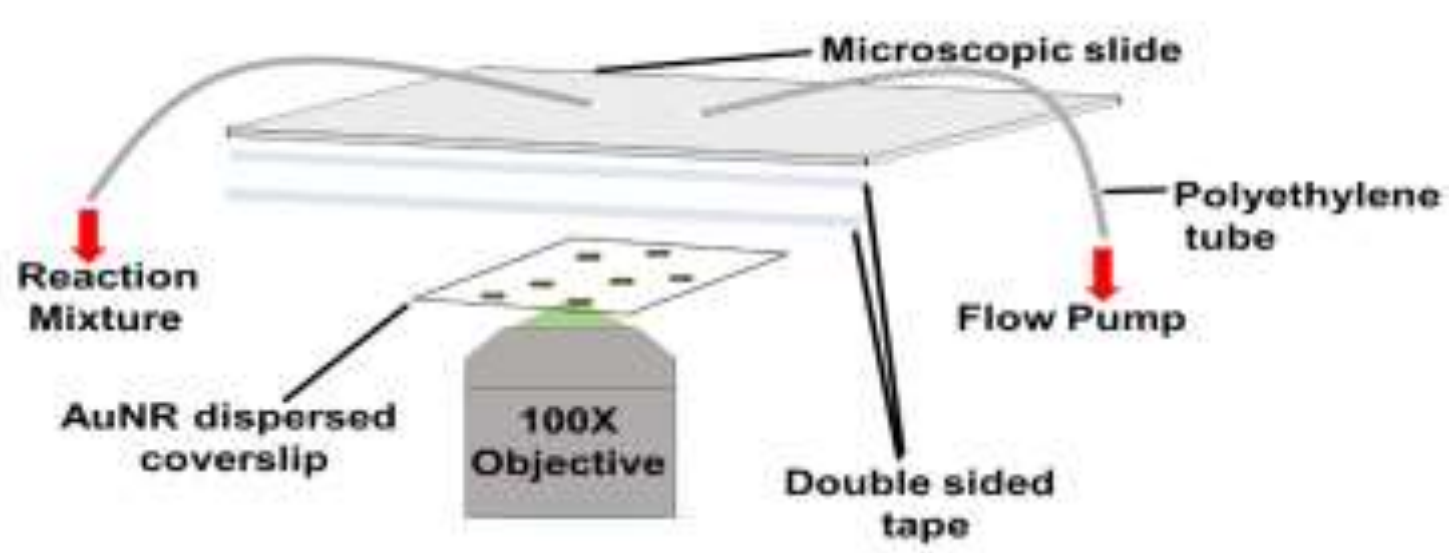

Figure S9. Schematic of the single-particle TIRF microscopy setup for AuNR@DNA coronazyme catalysis measurements, showing the 532 nm excitation laser, external LED source for LSPR excitation, microfluidic flow cell, and sCMOS detection system.

S7.2 Single-Molecule Data Processing

Fluorescence images were analyzed using ThunderSTORM,[S8] an ImageJ plugin, in combination with a custom-written Matlab program, following our established procedure.[S7] Analysis consisted of three sequential steps.

First, raw fluorescence images were filtered using a B-spline wavelet algorithm, and individual RF molecules in each frame were identified as local intensity maxima. The centroid position of each molecule was determined by fitting its emission profile to a two-dimensional Gaussian point-spread function:

$$PSF_G(x, y \mid \theta) = (\theta_N / 2\pi\, \theta_\sigma^2) \exp[-((x - \theta_x)^2 + (y - \theta_y)^2) / 2\theta_\sigma^2] + \theta_b \qquad \text{(S7)}$$

where θ_x and θ_y are the sub-pixel centroid coordinates, θ_σ is the fitted width of the emission spot, θ_N is the total photon count, and θ_b is the background offset. Fitted localizations were filtered by localization uncertainty (3 to 40 nm) and σ value (20 to 500 nm) to reject signals inconsistent with single fluorophores.

Second, localized RF centroids were assigned to individual AuNR@DNA coronazymes using a nearest-neighbor algorithm implemented in Matlab. Each coronazyme's position and the frame index of every associated RF detection event were recorded. Super-resolution localization (~4 nm precision) was assigned to each single-particle fluorescence bursts their respective nanoparticles by grouping the RF emission signal centroids within a 200 nm × 200 nm region. Any catalytic hotspots that were excessively large were excluded since they could indicate dimers, multimers, or huge aggregates.

Third, the frame indices of RF events on each coronazyme were used to extract two characteristic waiting times: τ_{off}, the dark interval between two consecutive RF bursts on the same particle, which reflects the catalytic turnover time; and τ_{on}, the duration of each fluorescence burst, which reflects the RF residence time. Because τ_{on} was short (1–3 frames) relative to τ_{off} under our experimental conditions, the combined waiting time $\tau = \tau_{off} + \tau_{on}$ was used as the single-turnover time. The time-averaged turnover rate of each individual coronazyme was calculated as τ^{-1}.

S7.3 Modified Michaelis-Menten Kinetics

Standard Michaelis-Menten kinetics describes enzymes with a single active site. Because AuNR@DNA coronazymes present multiple reactive sites within the DNA corona, a modified form of the Michaelis-Menten equation was applied:

$$v = V_{\max} [S] / (K_M + [S]) \qquad (S8)$$

where $V_{\max}$ is the highest reaction rate per coronazyme, K_M is the Michaelis-Menten constant, and [S] is the AR concentration. The reaction rate v on a single coronazyme equals τ^{-1}. Kinetic parameters were obtained by fitting the concentration-dependent turnover rate data to Eq. S3. For reference, parameters for the AuNS@DNA coronazyme reported previously[S7] are included for comparison.

Table S2. Kinetic parameters for citrate-capped AuNR and AuNR@DNA coronazymes, with AuNS and AuNS@DNA coronazyme values included for comparison.

**Parameter**	**AuNR**	**AuNR@DNA**	**AuNS**[a]	**AuNS@DNA**[a]
$V_{\max}$ (s^{-1})	0.050 ± 0.002	0.059 ± 0.001	0.031 ± 0.001	0.036 ± 0.001
K_M (μM)	0.24 ± 0.02	0.11 ± 0.01	0.21 ± 0.02	0.048 ± 0.002
$V_{\max}/K_M$ (s^{-1} μM^{-1})	0.21 ± 0.03	0.54 ± 0.06	0.15 ± 0.01	0.75 ± 0.03

[a] Reprinted in part with permission from Zuo et al., *J. Am. Chem. Soc.* 2023, 145, 5750–5758. Copyright 2023 American Chemical Society.

The catalytic efficiency of bare AuNR (0.21 ± 0.03 s^{-1} μM^{-1}) exceeds that of bare AuNS (0.15 ± 0.01 s^{-1} μM^{-1}), consistent with the larger surface area and greater number of reactive sites available on AuNRs. Upon DNA functionalization, the catalytic efficiency of AuNR@DNA (0.54 ± 0.06 s^{-1} μM^{-1}) is 2.6-fold higher than bare AuNR, reflecting the combined contributions of the DNA corona to substrate binding and long-range charge transport. The more modest enhancement observed for AuNR@DNA relative to AuNS@DNA (5-fold) is consistent with incomplete DNA coverage of the AuNR sidewall, which leaves exposed gold surface area contributing a less efficient direct-surface catalytic pathway.

S7.4 Reactivity Fluctuation Analysis

The surface of plasmonic nanoparticles undergoes continuous restructuring during catalysis, giving rise to fluctuating reactivity at the single-particle level, a phenomenon known as dynamic disorder.[S9, 10] This stochastic fluctuation is not random: the autocorrelation function of the single-particle waiting time series $\tau(t)$ exhibits a single-exponential decay, reflecting a catalytic memory effect governed by the timescale of surface restructuring. The inverse of the autocorrelation decay constant yields the reactivity fluctuation rate.

For both bare AuNRs and AuNR@DNA under dark conditions, the reactivity fluctuation rate increases linearly with the mean catalytic turnover rate, indicating that surface restructuring is driven by adsorbate-surface interactions, consistent with the behavior previously observed for AuNS and AuNS@DNA.[S7] The persistence of this linear dependence in AuNR@DNA, unlike the turnover-rate-independent fluctuations seen in the fully covered AuNS@DNA, confirms that exposed AuNR sidewalls contribute to the catalytic signal and surface dynamics across all DNA:AuNR feed ratios tested.

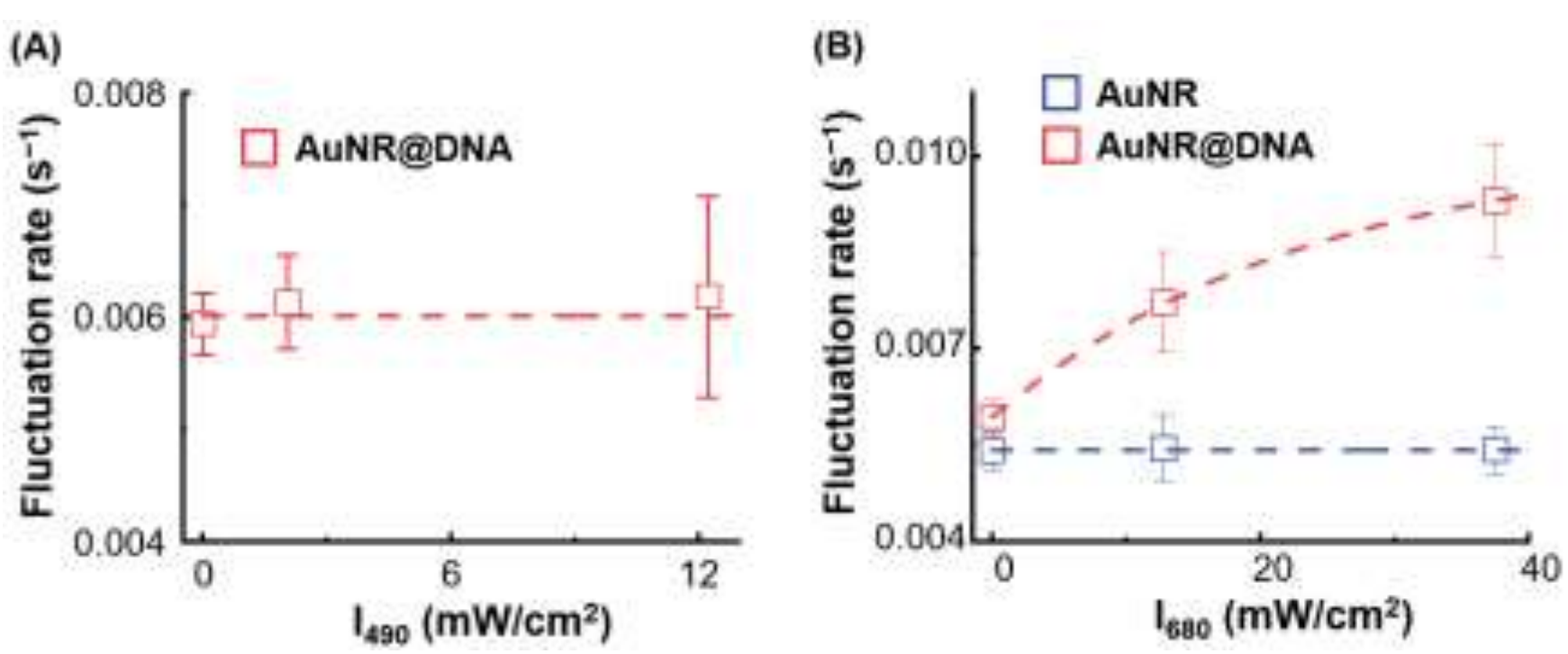

Figure S10. Dependence of reactivity fluctuation rate on LED power density for (A) AuNR@DNA under 490 nm illumination, (B) citrate capped AuNR under 680 nm illumination, AuNR@DNA under 680 nm illumination. [AR] = 800 nM for all experiments. Error bars represent s.e.m.; each data point is an average of more than 60 particles.

Under 680 nm LED illumination, the reactivity fluctuation rate of AuNR@DNA increases proportionally with light intensity (**Fig. S10B**), indicating that CID-mediated charge injection under longitudinal LSPR excitation accelerates surface dynamics in a photon-dependent manner.

This observation is consistent with enhanced charge transfer at the Au-DNA interface under 680 nm excitation, which increases the rate at which reactive intermediates are formed and consumed at the corona. In contrast, neither citrate-capped AuNRs under 680 nm illumination nor AuNR@DNA under 490 nm illumination show a light-intensity-dependent fluctuation rate (**Fig. S10A, B**), further confirming that the observed plasmon-enhanced dynamics are specific to the longitudinal LSPR excitation of AuNR@DNA and are not a photothermal artifact.

S7.5 Plasmon-Enhanced Coronazyme Catalysis: Experimental Protocol

For plasmon-enhanced catalysis measurements, the TIRF microscopy setup described in Section S7.1 was used with additional LED illumination. A 490 nm or 680 nm LED (Thorlabs Inc.) was mounted in the transmitted illumination path and directed vertically onto the reaction chamber (**Fig. S8**). LED power density was varied from 0 to 40 mW cm^{-2} (680 nm) or 0 to 15 mW cm^{-2} (490 nm) using a variable neutral density filter. All other acquisition and data processing parameters were identical to those described in Sections S7.1 and S7.2. For each power density, at least 60 individual particles were analyzed to obtain statistically reliable turnover rate distributions.

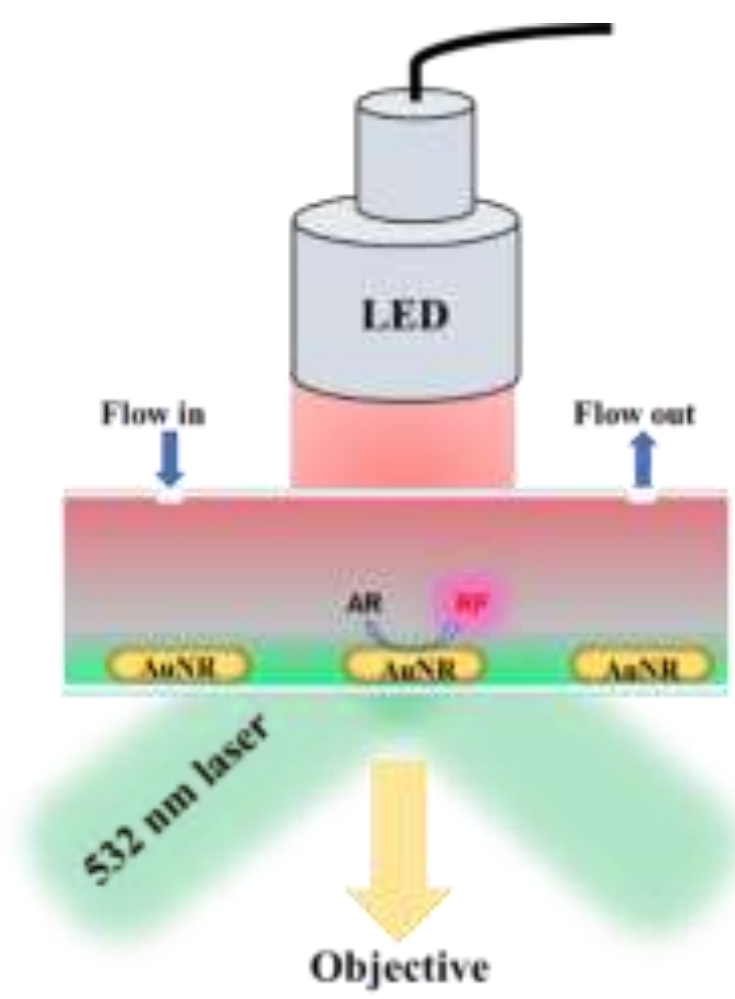

Figure S11. Schematic of the single-particle catalysis setup with LED illumination for plasmon-enhanced experiments, showing the 490 nm or 680 nm LED light path relative to the TIRF excitation geometry.

S7.6 SYBR Gold fluorescence quantification of DNA content on AuNRs

SYBR Gold produces a large fluorescence enhancement upon intercalation into DNA, enabling quantification of the DNA content associated with the AuNR. For SYBR Gold fluorescence quantification of DNA content on AuNRs, the TIRF microscopy setup described in Section S7.1 was used. Flow cell construction followed the same procedure in Section S7.1. The flow cell was continuously supplied with a ~5 nM SYBR gold solution, and the single molecule fluorescence microscopy setup was used to record. The average number of SYBR Gold fluorescence bursts per AuNR particle at varying feed ratios of 2:1, 10:1, 100:1 and 200:1 was 42.0 ± 1.5, 46.9 ± 1.9, 46.3 ±1.7 and 45.9 ± 2.3, respectively **(Fig. S12).** The error depicted represents s.e.m.. The results reveal a slight but consistent increase in the number of bound DNA molecules from the 2:1 to 100:1 feed ratios, after which the 200:1 condition shows no further increase relative to 100:1, indicating that the available binding sites on the AuNR surface became saturated at 100:1. Importantly, the absolute differences in bound DNA content across the 2:1, 10:1, and 100:1 conditions, while real, are modest compared to the order-of-magnitude variation in the feed ratio.

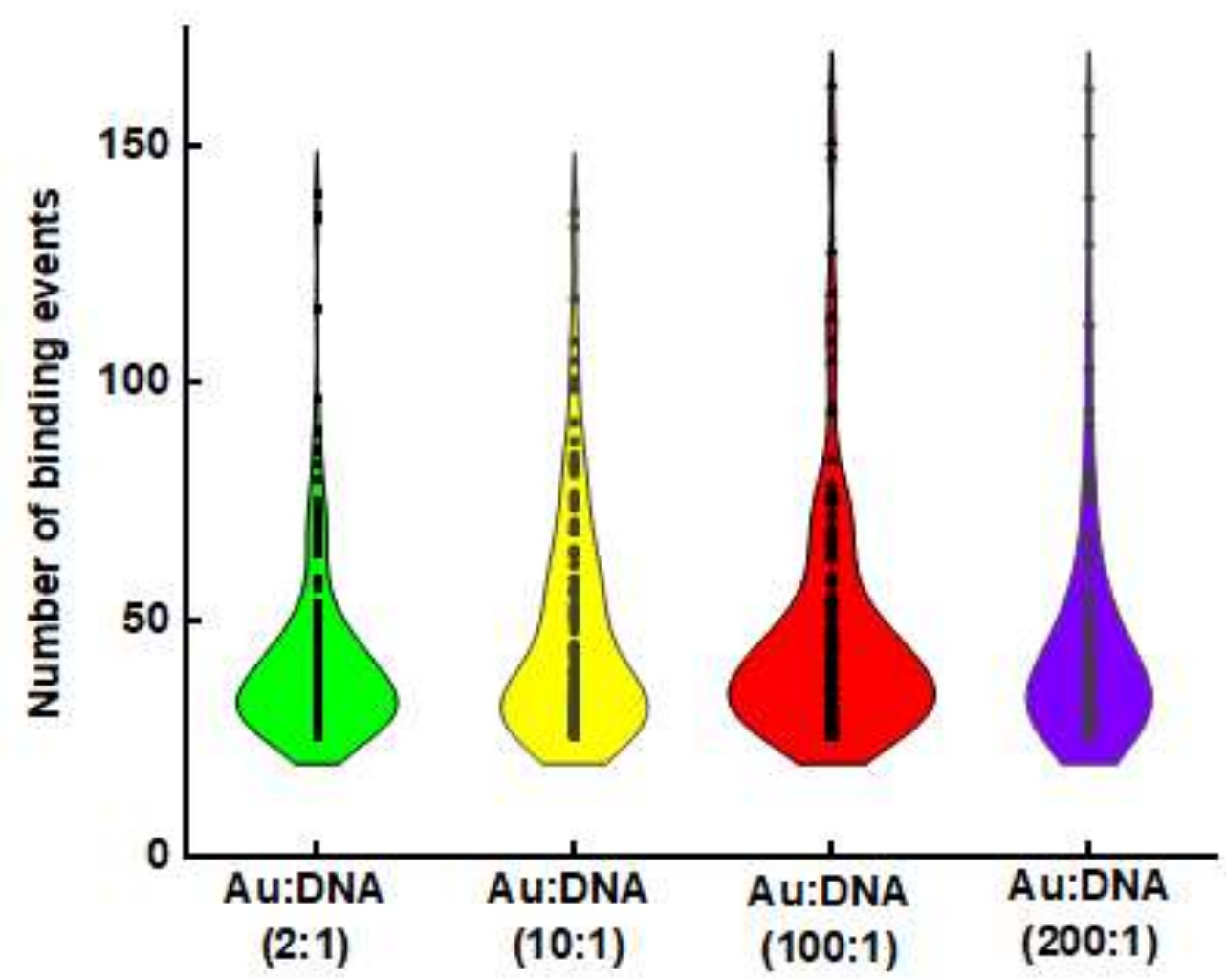

Figure S12. SYBR Gold fluorescence quantification of DNA content on AuNRs at varying DNA: AuNR feed ratios. Violin plots showing the distribution of SYBR Gold fluorescence bursts per AuNR particle at DNA:AuNR feed ratios of 2:1, 10:1, 100:1, and 200:1 (n > 100 particles per condition). Each data point represents the number of SYBR Gold fluorescence bursts detected on a single AuNR, reflecting the number of DNA molecules associated with that particle.

S8. Estimation of temperature rise on AuNR under photoexcitation

To estimate the temperature rise on AuNR due to the LED illumination, we first simulated the absorption spectrum of a single AuNR (18 × 52 nm) on a glass substrate in water using an MNPBEM package.[S11-13]

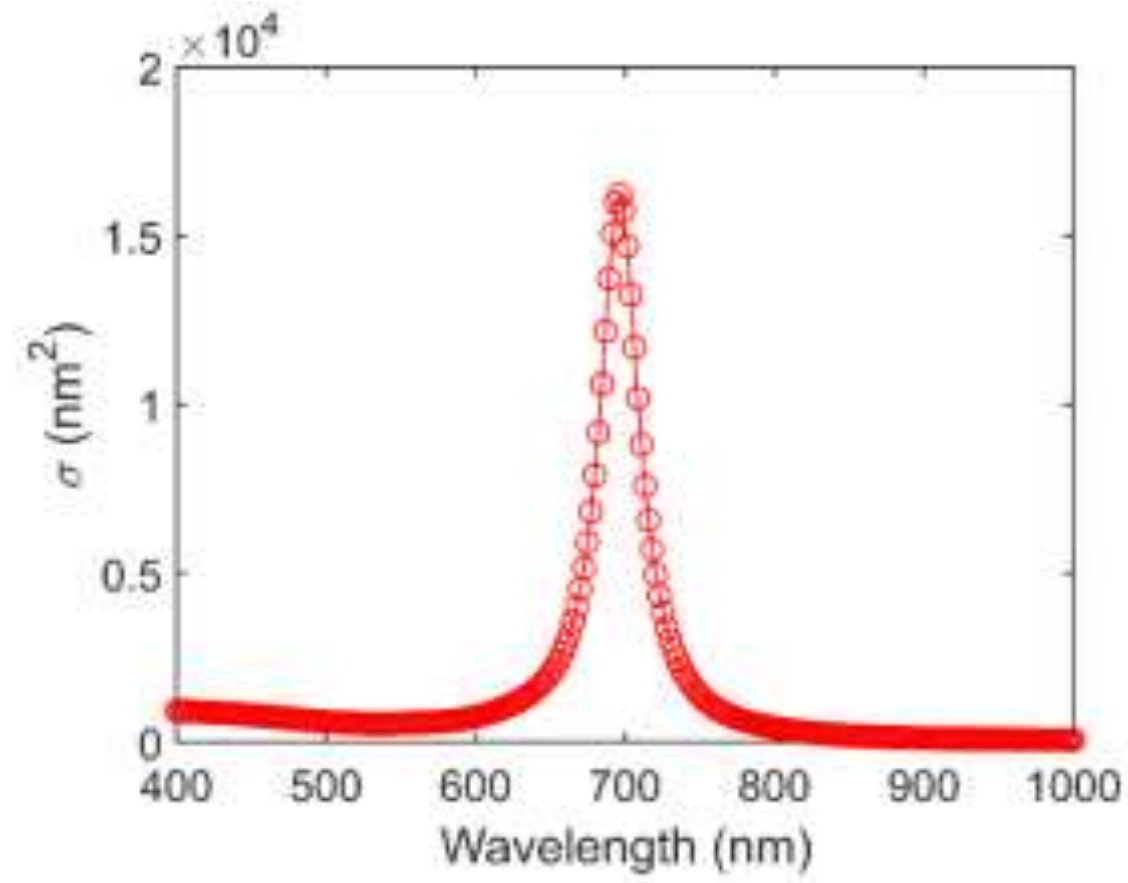

Figure S13. Simulated absorption spectrum of AuNRs (18 × 52 nm) on a glass substrate in water medium. The spectrum is the average of two spectra with excitation polarization parallel and perpendicular to the long axis of AuNR to mimic unpolarized LED illumination.

To estimate temperature rise (ΔT) on AuNR, the following equation by Baffou et al[S14] was used:

$$\Delta T = \frac{P_{\mathrm{abs}}}{4\pi\kappa\beta R_{\mathrm{eq}}} \qquad \text{(S9)}$$

$$P_{\mathrm{abs}} = I\,\sigma_{\mathrm{abs}} \qquad \text{(S10)}$$

where σ_{abs} is the absorption cross section of AuNR, I is the illumination power density of LED, κ is thermal conductivity, β is the shape factor, and R_{eq} is the equivalent radius of AuNR.

We have listed all the calculated and experimental parameters in the following table.

Table S3. Parameters for estimation of temperature rise on AuNR under photoexcitation.

$\boldsymbol{\sigma_{\mathrm{abs,\ 680\ nm}}}$ **(m²)**	$\boldsymbol{I_{680}}$ **(W m⁻²)**	$\boldsymbol{\kappa}$ **(W m⁻¹ K⁻¹)**	$\boldsymbol{\beta}$	$\boldsymbol{R_{\mathrm{eq}}}$ **(m)**
8.16×10^{-15}	400	0.606	0.93	12.82×10^{-9}

Therefore,

$$\Delta T_{680} = 3.59 \times 10^{-5}\, K$$

Due to the low LED power density, the estimated temperature rise is only 35.9 μK, indicating that photothermal heating is negligible under the experimental conditions.

Supporting Information References

(S1) Foerster, B.; Joplin, A.; Kaefer, K.; Celiksoy, S.; Link, S.; Sönnichsen, C. Chemical interface damping depends on electrons reaching the surface. *ACS nano* **2017**, *11* (3), 2886-2893.

(S2) Hoggard, A.; Wang, L.-Y.; Ma, L.; Fang, Y.; You, G.; Olson, J.; Liu, Z.; Chang, W.-S.; Ajayan, P. M.; Link, S. Using the plasmon linewidth to calculate the time and efficiency of electron transfer between gold nanorods and graphene. *ACS nano* **2013**, *7* (12), 11209.

(S3) Lee, J.; Ha, J. W. In Situ Photoreversible Tuning of Chemical Interface Damping in Single Gold Nanorods Through Cucurbit [8] uril-Based Host–Guest Interactions. *ACS Applied Materials & Interfaces* **2024**, *16* (34), 45763-45770.

(S4) Vo, T.; Chang, W.-S. Plasmon-induced electron transfer between gold nanorods and a carbon thin film. *The Journal of Physical Chemistry C* **2023**, *128* (1), 321-329.

(S5) Lee, J.; Ha, J. W. Effects of amine linkers with different carbon chain lengths at guanine-rich polynucleotides on chemical interface damping in single gold nanorods. *Analytical chemistry* **2022**, *94* (19), 7100-7106.

(S6) Foerster, B.; Rutten, J.; Pham, H.; Link, S.; Sönnichsen, C. Particle plasmons as dipole antennas: State representation of relative observables. *The Journal of Physical Chemistry C* **2018**, *122* (33), 19116-19123.

(S7) Zuo, L.; Ren, K.; Guo, X.; Pokhrel, P.; Pokhrel, B.; Hossain, M. A.; Chen, Z.-X.; Mao, H.; Shen, H. Amalgamation of DNAzyme and Nanozyme in a Coronazyme. *J. Am. Chem. Soc.* **2023**, *145* (10), 5750.

(S8) Ovesný, M.; Křížek, P.; Borkovec, J.; Švindrych, Z.; Hagen, G. M. ThunderSTORM: a comprehensive ImageJ plug-in for PALM and STORM data analysis and super-resolution imaging. *Bioinformatics* **2014**, *30* (16), 2389-2390.

(S9) Lu, H. P.; Xun, L.; Xie, X. S. Single-molecule enzymatic dynamics. *Science* **1998**, *282* (5395), 1877-1882.

(S10) Xu, W.; Kong, J. S.; Yeh, Y.-T. E.; Chen, P. Single-molecule nanocatalysis reveals heterogeneous reaction pathways and catalytic dynamics. *Nature materials* **2008**, *7* (12), 992-996.

(S11) Hohenester, U. Simulating electron energy loss spectroscopy with the MNPBEM toolbox. *Computer Physics Communications* **2014**, *185* (3), 1177-1187.

(S12) Hohenester, U.; Trügler, A. MNPBEM–A Matlab toolbox for the simulation of plasmonic nanoparticles. *Computer Physics Communications* **2012**, *183* (2), 370-381.

(S13) Waxenegger, J.; Trügler, A.; Hohenester, U. Plasmonics simulations with the MNPBEM toolbox: Consideration of substrates and layer structures. *Computer Physics Communications* **2015**, *193*, 138-150.
(S14) Baffou, G.; Quidant, R.; García de Abajo, F. J. Nanoscale Control of Optical Heating in Complex Plasmonic Systems. *ACS Nano* **2010**, *4* (2), 709-716. DOI: 10.1021/nn901144d.
